# Facilitators and barriers of implementation of routine postnatal care guidelines for women: A systematic scoping review using critical interpretive synthesis

**DOI:** 10.7189/jogh.13.04176

**Published:** 2023-11-24

**Authors:** Lenka Beňová, Aline Semaan, Anayda Portela, Mercedes Bonet, Thomas van den Akker, Andrea B Pembe, Allisyn Moran, Diane Duclos

**Affiliations:** 1 Institute of Tropical Medicine, Antwerp, Belgium; 2 London School of Hygiene and Tropical Medicine, London, UK; 3 Vrije Universiteit Amsterdam, Amsterdam, the Netherlands; 4 World Health Organization, Department of Maternal, Newborn, Child and Adolescent Health and Ageing, Geneva, Switzerland; 5 UNDP/UNFPA/UNICEF/WHO/World Bank Special Programme of Research, Development and Research Training in Human Reproduction (HRP), Department of Sexual and Reproductive Health and Research, World Health Organization, Geneva, Switzerland; 6 Leiden University Medical Centre, Leiden, the Netherlands; 7 Muhimbili University of Health and Allied Sciences, Dar es Salaam Tanzania (s)

## Abstract

**Background:**

Postnatal care (PNC) has the potential to prevent a substantial burden of maternal and newborn morbidity and mortality. This scoping review aimed to identify and synthesise themes related to facilitators and barriers of implementation of guidelines on routine PNC for women (postpartum care) in all settings.

**Methods:**

This is a scoping review guided by the standard principles of Arksey & O’Malley’s framework. We used the critical interpretive synthesis method to synthesise the whole body of evidence. We searched four databases (Medline, Embase, Global Health, CINAHL Plus) using a combination of search terms comprising four key concepts: postnatal care, routine care, guidelines and implementation. No restrictions on country or language of publication were applied. We excluded studies not presenting findings about PNC for women. We thematically charted the themes of studies included based on title and abstract screening. All studies included after full text screening were described and their results synthesised using the socio-ecological model framework. We did not conduct a risk of bias analysis or quality assessment of included studies.

**Results:**

We identified a total of 8692 unique records and included 43 studies which identified facilitators and barriers to implementing routine guidelines in provision of PNC to women. Three quarters of studies pertained to PNC provision in high-income countries. Specific facilitators and barriers were identified and thematically presented based on whether they affect the provision of PNC or the intersection between provision of PNC and its use by women and families. We applied a critical global health lens to synthesise three constructs in the literature: finding a balance between standardisation and individualisation of PNC, the fragmented PNC provision landscape complicating the experiences of women with intersecting vulnerabilities, and the heavy reliance on the short postpartum period as an opportunity to educate and retain women and newborns in the health system.

**Conclusions:**

This interpretive synthesis of evidence shows that the fragmented and narrow nature of PNC provision presents specific challenges to developing, adapting and implementing routine PNC guidelines. This results in a lack of linkages to social support and services, fails to address intersecting vulnerabilities and inequities among women, and negatively influences care seeking. There is a lack of evidence on how processes of individualising PNC provision can be applied in practice to support health workers in providing woman-centered PNC in various global settings.

**Registration:**

https://www.protocols.io/private/C99DA688881F11EBB4690A58A9FEAC02

Although substantial progress has been made to meet the Sustainable Development Goals (SDGs), maternal and perinatal mortality remain unacceptably high in most low- and middle-income countries (LMICs). Globally, progress in reducing maternal mortality has stalled or declined in 2020 [1]. The latest estimates indicate that 287 000 maternal deaths occurred in 2020, 2.5 million newborns died in 2018 and 1.9 million stillbirths occurred in 2019 [1,2]. Tackling this burden has been prioritised in national, regional and global actions, with ambitious targets set for maternal and newborn survival and well-being [3,4]. Maternal health related targets of the SDGs include reducing the global maternal mortality ratio to less than 70 per 100 000 live births and reducing neonatal mortality to less than 12 per 1000 live births by 2030 [5]. Preventive, curative and health promotion interventions that improve maternal and newborn survival include improving of health care coverage and contacts with the care system. However, despite rising proportions of women seeking skilled care during pregnancy, childbirth and the postnatal period, maternal and neonatal mortality rates are 10-40-fold higher in the most affected region (Sub-Saharan Africa) compared to high-income settings such as Europe and North America [1,6,7]. This is largely due to difficulties in the implementation of comprehensive, continuous, evidence-based care along the continuum [8].

The largest burden of maternal and perinatal deaths occurs during labor, childbirth and the immediate postnatal period, followed by the extended postnatal period [9,10]. The most important causes of postpartum maternal deaths are hemorrhage, sepsis, hypertensive diseases of pregnancy and indirect causes such as human immunodeficiency virus (HIV) and malaria. Between 20 and 44% of maternal deaths in Sub-Saharan Africa – region with the highest maternal morality ratio – are estimated to occur in the postnatal period [11,12]. The vast majority of these deaths are preventable with timely access to good quality care; improving postnatal care (PNC) has the potential to prevent about 60% of maternal deaths (i.e. saving >150 000 lives per year) [13]. The extended postnatal period, beyond the 42-day time frame covered in traditional PNC models [14] is also a determinant for the longer-term health and well-being of women and children and can be leveraged to promote family planning use, continued exclusive breastfeeding and bonding between woman and infant, among others. Postnatal care is therefore an essential component of the continuum of care for maternal and newborn health [15,16]. In this study we focus on postpartum care for women, but because this is inextricably linked to care for the newborn, we use the broader term for the period (postnatal).

Despite increasing proportions of women seeking antenatal and childbirth care globally, PNC coverage remains low [17] and this is the case even among women who give birth in health facilities. A study using data from 33 Sub-Saharan African countries found that almost one third of women left the facility where they gave birth without receiving a postnatal check there [18]. Globally, racial and ethnic disparities affect access to postnatal services [19,20]. In LMICs, the use of PNC is inequitable and varies according to socio-economic status and between urban and rural settings [21]. Critical gaps exist on the supply side in terms of the availability of several elements of PNC, including lack of screening and counselling or support for mental health and domestic abuse [22] and social protection [23]. From the demand side, women report concerns around access to and quality of PNC, and low perceived value of PNC in terms of health benefits for healthy women and newborns, with specific needs highlighted for adolescent mothers, such as tailored health and social services to support them as they continue their education and professional trajectories [24,25]. Family members, especially fathers/partners, express the need for more information, care and psychological support, which are not being met in the traditional model of PNC, and hinder the accessibility and acceptability of PNC [26]. Additionally, the quality of antenatal and intrapartum care influences women’s and families’ care seeking patterns postnatally [27,28].

The World Health Organization (WHO) defines a positive postnatal experience “as one in which women, newborns, partners, parents, caregivers and families receive information, reassurance and support in a consistent manner from motivated health workers; where a resourced and flexible health system recognises the needs of women and babies, and respects their cultural context” [29,30]. In 2022, the WHO updated the guidelines to improve facility-based and community-based PNC, confirming the recommendation of a minimum of four postnatal care contacts up to six weeks after birth [29]. The content of care for the woman includes physiological assessments and interventions, screening and prevention for postpartum depression and anxiety and nutritional and physical activity recommendations, and counselling on contraceptive measures. Considering the large variability in platforms designated to meet the needs of postpartum women, ranging from health facilities, outpatient visits, to home visits, the WHO emphasises the need to adapt the recommendations to local settings [29]. Globally, PNC guidelines are formulated and implemented with large variability, and different factors influence their implementation at the individual, family, societal, health system, and policy level.

This scoping review aims to identify and synthesise themes related to facilitators and barriers of implementation of guidelines on routine postnatal care for women in all global settings as captured in published and gray literature.

## METHODS

This scoping review was guided by the standard principles of Arksey & O’Malley’s framework and the PRISMA-ScR checklist (S1 in **Online Supplementary Document**) [31,32]. Arksey & O’Malley’s approach can be described as an iterative process involving post-hoc inclusion and exclusion criteria. According to this framework there are five stages: 1) identifying the research question, 2) identifying relevant studies, 3) study selection, 4) charting the data, and lastly 5) collating, summarising and reporting the results. In addition, we use a method called “critical interpretive synthesis” [33], which enables us to apply a qualitative interpretive approach to the whole body of evidence. This is an inductive approach which begins with a wide question and narrows the scope during the process of screening (mapping of themes and study types), before arriving at full text review and examination of broad questions such as how the available literature constructs its problematics and the nature of the assumptions [33]. It has been successfully applied in studies of maternal health, for example for continuum of care [34]. The protocol for this scoping review was published on protocols.io on 23 March 2021 [35].

### Definitions

Postnatal care refers to the provision of care after birth to the woman, newborn(s), or both. A common definition of the postnatal period is the first 42 days after childbirth or six weeks since the birth. However, longer periods of time might be specified in postnatal care guidelines, in which case we were guided by these. This review’s focus on PNC specifically for women aims to cover various perspectives from which this topic is captured in the literature, including the broader health system, specific health facilities, individual health workers, and community-based provision of postnatal care. However, studies focusing exclusively on recipients’ (women’s and families’) experiences of postnatal care were not the focus of this review, as a separate literature assessing this dimension was published recently [24]. In addition, a review on health workers’ perspectives is being conducted [36].

Routine postnatal care refers to elements of care performed by a health worker within the formal health system to deliver screening, preventive interventions, counselling/health education or support and routine referral pathways which are to be provided or in place for every woman. Multiple elements of care which are supposed to be provided together or in combination are referred to as a “package of care”. The specific focus of this review is to understand how the routine postnatal care elements and the modality of their provision (number of contacts, their timing, type of health worker, platform for care provision) as described in the formal guidelines issued by a national or sub-national health authority are implemented by actors within the formal health system (health providers and health facilities), regardless of the location of birth (in domestic environment or in health facilities).

The term “guidelines” (or “clinical practice guidelines”) in this review refers to formal documents and practices prescribing the content, type, number and timing of various elements of care which are to be provided by health systems, facilities and health workers to women. This includes documents used at health facility level such as standard operating procedures, nursing instructions, checklists, as well as national care guidelines which could be in the form of documents issued by Ministries of Health, health insurance companies or professional associations.

### Searches

We searched four databases of peer-reviewed literature (Medline, Embase, Global Health, CINAHL Plus) using a combination of search terms comprising four key concepts: postnatal care, routine care, guidelines, and implementation (complete search terms are in S2 in the **Online Supplementary Document**). Searches were conducted on 23 March 2021 and restricted to studies published since the year 2000 to focus on contemporary evidence. No restrictions on country or language of publication were applied.

### Study selection

We conducted study selection in three steps, allowing us to chart the themes appearing in the literature as we narrowed into the research objective of this paper. In the first step, we imported the database search results into EndNote and deduplicated the records [37]. This deduplicated list of references was imported into Rayyan, where two people (LB and AS) conducted a screening based on title and abstract independently. In this step, we included manuscripts published in peer-reviewed journals or reports, if they reported findings from quantitative and qualitative studies, systematic reviews of the evidence, original analysis of secondary evidence, modeling analysis or cost-effectiveness studies. All studies which mentioned facilitators or barriers to routine postnatal care guideline implementation using any method (qualitative, quantitative, or mixed) were included, which meant those studies having an observational or intervention design, as well as reviews summarising evidence from the literature. We excluded conference abstracts and thesis documents. We excluded studies not reporting on women, meaning those reporting on newborns only. Given the timing of the search, coronavirus disease 2019 (COVID-19) specific papers would have been considered eligible; however, no such studies were identified. The second step involved thematically charting the themes of the remaining studies based on title and abstract and applying inclusion and exclusion criteria in full text screening. All records were double-coded by LB and AS based on detailed criteria shown in S3 in **Online Supplementary Document**. This process allowed us to further describe the themes appearing in the broader literature on postnatal care at the same time as identifying studies to include in this review. In step three, all retained studies were reviewed in full text by LB, AS and DD.

### Charting the data

We constructed a data extraction table for studies included in full text review containing the following items: (a) study design and year of publication; (b) country, country income group, and context (geographic area; health care facility type); (c) study method (e.g. qualitative, quantitative, mixed); (d) sample characteristics (sample size, population (women, health workers, etc.)); (e) guideline characteristics (national/facility level, location/platform of PNC provision, type of provider, content (single element or package of care), and recommendations); (f) other elements of the assessment of implementation; and (g) facilitators/barriers identified by the study. Data extraction was conducted by LB, AS and DD in Microsoft Excel. We did not conduct a risk of bias analysis or quality assessment of included studies as this is not usually a part of scoping reviews or reviews using critical interpretive synthesis.

### Role of reflexivity in collating, summarising and reporting the results

Critical interpretive synthesis [33] builds on the diversity of relevant studies to develop categories under which data can be summarised. This approach uses a reflective stance which recognises the value of expertise brought by authors to critique constructs emerging from studies. It therefore considers the authors’ influence on reporting findings and generating theoretical insights not as bias but as a strength for critical reinterpretation, as long as transparency and reflexivity are documented along this iterative process from data extraction, to data reporting, and critical analysis.

Upon completion of data extraction from included studies, we conducted three iterations of data synthesis. LB, AS and DD each synthesised information from a subset of included studies, classifying information according to the characteristics of the studies included, the themes emerging from the data and facilitators and barriers to the implementation of PNC guidelines listed in the different studies. To allow for iterative analysis, the three team members met between the three iterations of synthesis to discuss the classification process. Disciplinary expertise in public health and medical anthropology informed the categorisation process and synthesis discussed. Following the critical interpretive synthesis approach, we also used these discussions as an opportunity to question categories and assumptions found in the studies. Preliminary findings were then discussed with MB and AP to assess relevance embracing expertise from global health practice in the field of PNC guidelines.

### Integrative synthesis

We used the Socio-Ecological Model (SEM) to identify various levels of interrelated factors influencing the implementation of PNC guidelines inductively [38]. It also supports the synthesis and interpretation of data at the different levels, ranging from behavioral considerations to interpersonal and environmental factors. SEM was used to elicit a comprehensive synthesis and situate behavioral patterns in broader structures and interpersonal processes. This was done explicitly to recognise that health worker and health system factors are two of the many factors influencing the facilitators and barriers of routine postnatal care guideline implementation; the most important of which is the postpartum woman and her immediate social environment. Finally, in the interpretation, we drew on critical approaches to health system to inform a conceptual reframing of PNC on new constructs having emerged from this body of literature [39,40]. Combining these approaches enabled to identify facilitators and barriers to implementation of routine postnatal care guidelines, to recognise women’s preferences, needs and vulnerabilities, to situate these facilitators and barriers within complex and sometimes fragmented systems of provision, and to reflect on how to better integrate the diversity and sociality of PNC platforms in operational guidelines.

## RESULTS

### Searches and flowchart

**Figure 1:**
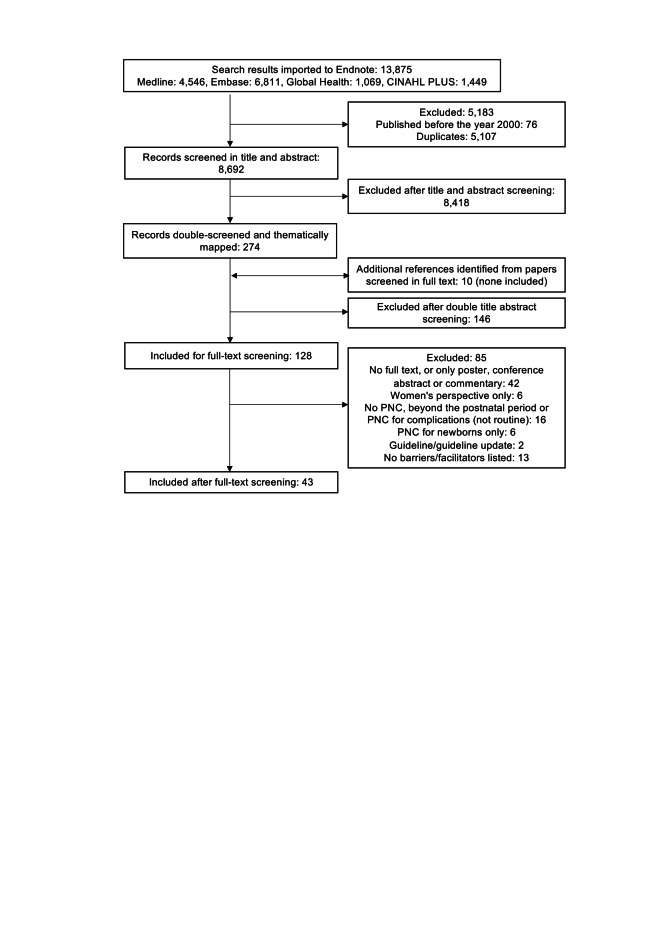
Study inclusion flow diagram.

**Figure 1** shows the steps of the searching and study inclusion process. We identified a total of 13 875 records by searching the four databases. We excluded 76 records published before 2000 and 5107 duplicate records. The remaining 8692 records were screened based on title and abstract and 8418 records were excluded. From the 274 records included for step two thematic assessment, 10 additional references were identified from reference lists; none were included in the review. The themes of the 274 remaining records were mapped on the basis of abstracts in the process of applying the inclusion and exclusion criteria. The full text screening of the remaining 128 records resulted in 43 studies being included in this review. We also mapped broad thematic areas appearing in records excluded at each step to construct a basic “eco-system” of the body of literature on postnatal care (S4 **in** **Online Supplementary Document**).

### Description of included studies

We extracted data from the forty-three included studies. A summary of characteristics of these studies is shown in **Table 1** and the full extraction sheet with details of each study in S5 in **Online Supplementary Document**. Most included studies were published since 2010 (86%) and pertained to PNC provision in high-income countries (74%). Three study designs were represented in the 43 studies: observational, intervention and reviews. In terms of the setting of PNC provision covered in the studies, two fifths (18/43) were from hospitals and 15 captured multiple locations where PNC is provided. In terms of the content of PNC, 22/43 studies tackled one element of routine PNC; 11 of which specifically discussed perinatal mental health and 21 studies were about package of multiple PNC elements. Three studies captured routine PNC for a sub-population of women.

**Table 1:** Characteristics of included studies (n = 43)

Characteristics	Number of studies
**Period of publication**	
2003-2009	6
2010-2021	37
**Country income group (World Bank 2021)**	
High income	32 (Australia: 10, United Kingdom: 8, USA: 6, Canada, Finland, France, Italy, Norway, Switzerland: 1 each, multi-country: 1)
Upper-middle income	1 (South Africa)
Lower-middle income	5 (Tanzania: 2, Ghana, Nigeria, Zimbabwe: 1 each)
Low-income	1 (Afghanistan)
Multiple countries	4
**Study design and methods**	
Intervention study (n = 15)	
*Qualitative*	2
*Quantitative*	8
*Mixed*	5
Observational study (n = 22)	
*Qualitative*	7
*Quantitative*	8
*Mixed*	7
Other (n = 6)	
*Literature review*	4
*Guideline review/evidence synthesis*	2
**Location of postnatal care provision**	
Single level/setting (n = 29)	
*Community, home visits*	3
*Primary care facility*	7
*Hospital*	18
Multiple settings (n = 15)	
*Community and primary care facility*	4
*Hospital and community*	2
*Other multiple settings*	9
**Postnatal care content**	
One element of routine postnatal care (n = 22)	
*Perinatal mental health*	11
*Breastfeeding/baby-friendly approaches/ parent-infant bonding*	4
*Care related to diabetes*	3
*Postnatal debriefing/other*	4
Package of routine postnatal care (n = 21)	
*For a sub-population of women/families*	3
*Secondary review of guidelines*	2
Assessment of adherence to guidelines in:	
*Primary care facility/community setting*	7
*Hospital setting/pre-discharge*	7
*Multiple care levels/settings*	2

### Summary of facilitators and barriers in included studies

The synthesis using SEM framework led us to identify five levels of socio-ecological influences, starting with the woman, newborn and the family, the individual health worker involved in PNC provision (e.g. midwife, obstetrician, general practitioner), the team of health workers, organisational level on a health facility or other levels, and finally the broader health system, policies and models within which PNC guidelines are defined, embedded and provided. By charting the specific facilitators and barriers extracted from the included studies, we developed two key themes: 1) provision of PNC, and 2) the intersection between provision of PNC and its use by women and families.

**Figure 2:**
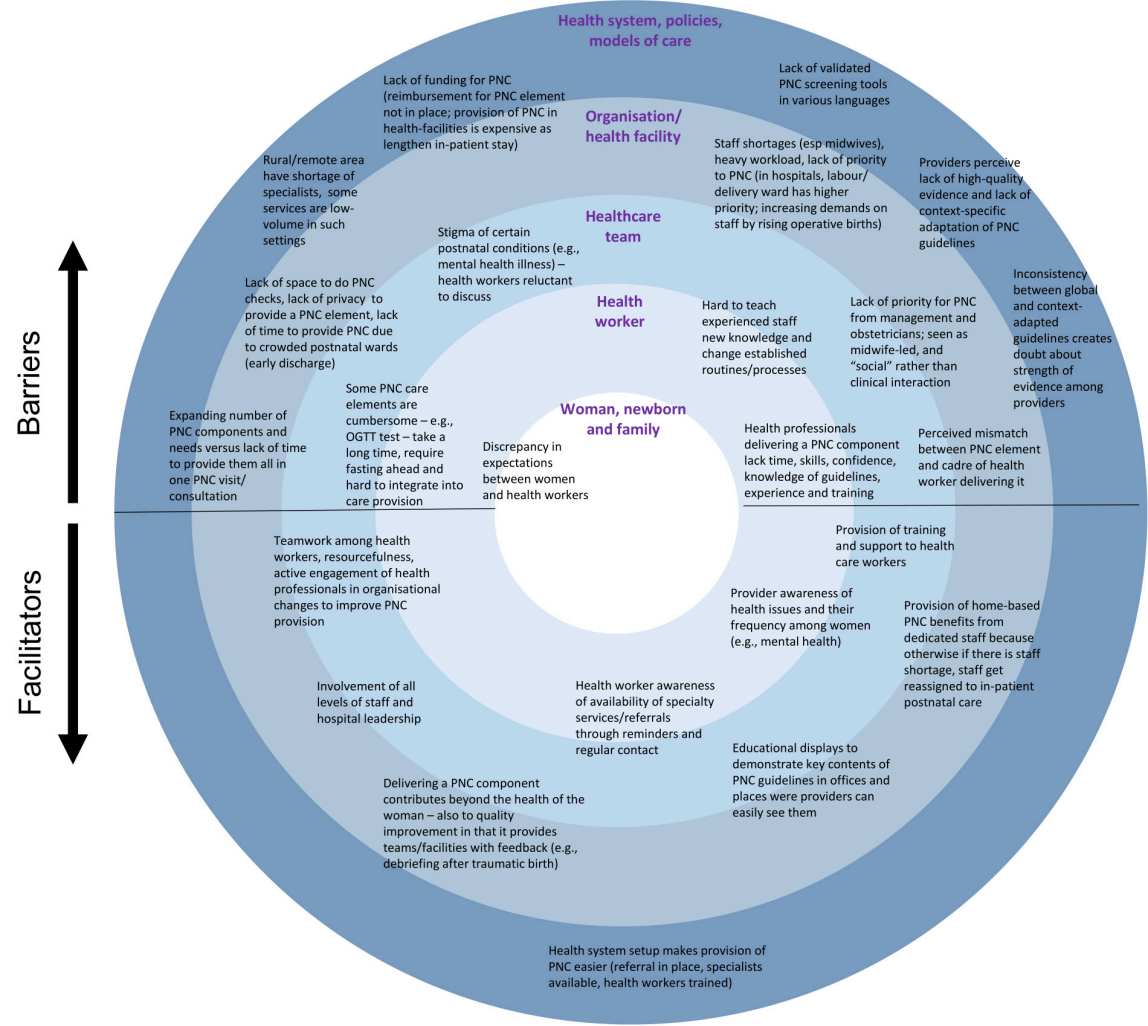
Facilitators and barriers of implementing routine postnatal care (PNC) guidelines for women related to the provision of PNC.

The first theme was related to the manner in which the provision (supply) of PNC is incorporated into the larger health system (**Figure 2**). The facilitators within this theme were related to actions and programmes aiming to increase the empowerment of health workers and to ensure sufficient staffing and training of health workers and teams. We note that while the barriers tended to be on all five levels of the SEM framework, the facilitators tended to cluster on the lower-levels and address issues related to health workers, teams and health facilities, rather than health systems and policies. The most important barriers were those capturing health workers’ perceptions that current PNC guidelines fall short in terms of context-adaptation and providing evidence for single care elements, which co-exists with low priority to PNC within the provision of health care services in general. This lack of priority is exemplified by insufficient time (e.g. within a consultation), attention, knowledge, awareness, staffing, space, supplies and tools afforded for PNC provision.

**Figure 3:**
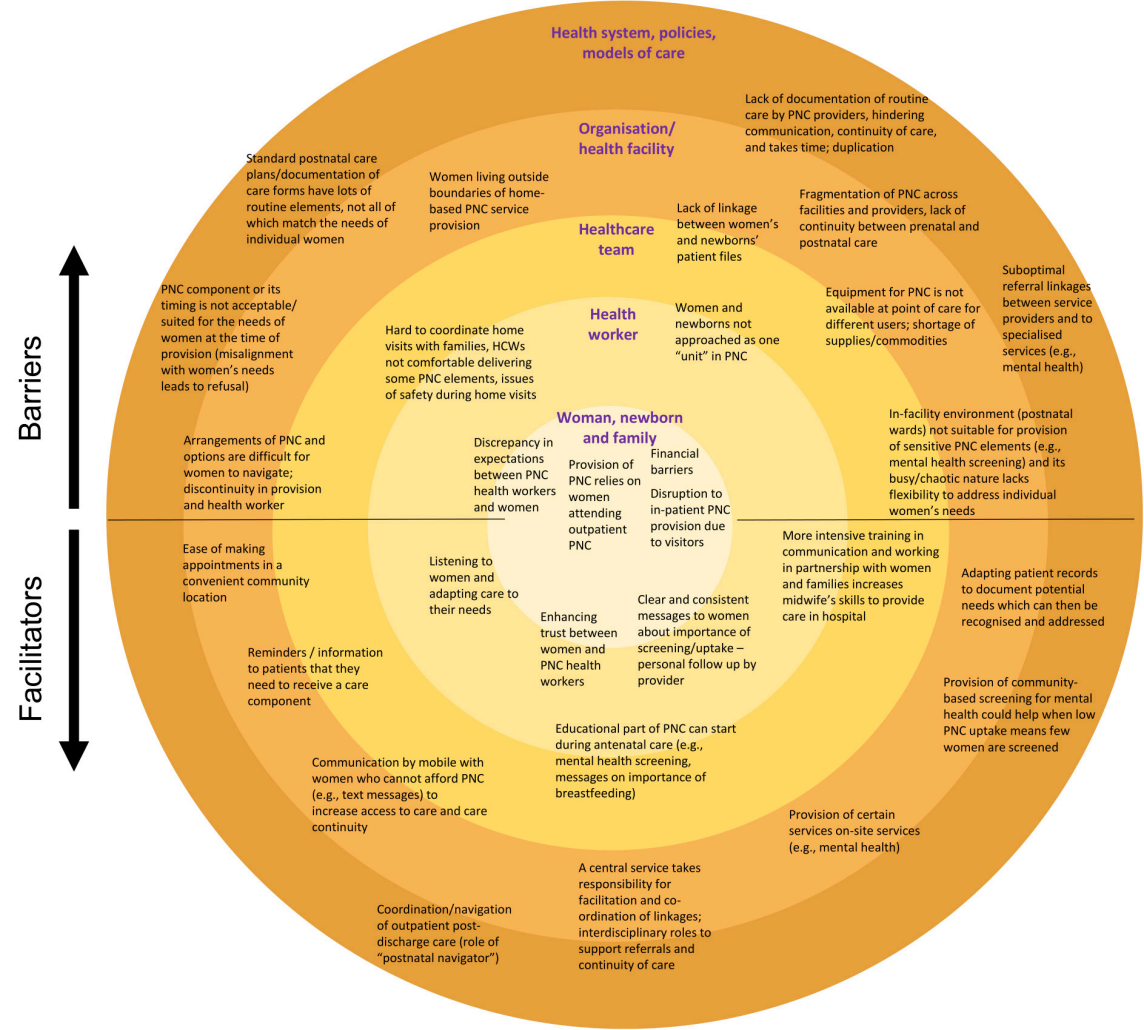
Facilitators and barriers of implementing routine postnatal care (PNC) guidelines for women related to the intersection between the provision and use of PNC.

The second theme (**Figure 3**) captured the intersection between provision of PNC and its use by women and families. Facilitators and barriers were identified on all levels of the SEM framework. Barriers commonly related to the lack of alignment between the needs of women and families and PNC guidelines or modalities of care provision. For example, lack of utilisation of (particularly) outpatient PNC by women has critical implications for the ability to deliver some routine PNC elements. Misalignment between women’s needs and the provision of PNC can also arise when an element of PNC is offered by someone other than women’s provider of choice, or at a timing not preferred by women and families, or when the system of PNC is complicated for women to navigate. We identified a large sub-theme capturing challenges with achieving continuity of postnatal care and leading to fragmentation of care. This included discontinuities between care for the woman and newborn(s), handovers between various cadres of health workers, facilities and platforms for care provision (e.g. referral process, discharge from facility to community-based care), across time (pregnancy to postpartum period) and within data systems (e.g. patient records). Facilitators of routine PNC provision according to guidelines included increasing navigability of PNC provision, for example through enhanced communication in the form of reminders, use of a postnatal navigator or central coordination of care linkages. Improvements in ability of health workers to tailor the provision and timing of PNC elements to meet individual women’s needs were also identified as key facilitators.

### Summary of cross-cutting themes influencing PNC guideline implementation

We extracted and analysed information that goes beyond facilitators and barriers to identify three cross-cutting themes influencing PNC guideline implementation across the different SEM framework levels. We describe them here and illustrate with examples from included studies. These three themes are closely inter-related and we explore how they build on each other.

#### Theme 1. PNC between standardisation and individualisation of care

Our review highlighted tensions between “consistency” and “flexibility” in PNC. On one hand, standardised, routine approaches (i.e. provision to every woman) and tools such as checklists allow to establish and offer common standards for PNC. Such approach might be more relevant in the immediate postpartum period where life-threatening complications might arise and detection and treatment of these issues must be assured consistently for every woman, keeping in mind that provision of guideline-congruent care can be hindered by a wide range of factors, such as health workers’ comfort and skills in addressing specific elements of care [41]. On the other hand, approaches to PNC increasingly promote individualised principles centered around women’s and families’ needs and preferences, particularly beyond the first hours and days after the birth. Even within the time spent in health facilities immediately after birth, routine elements specified in PNC guidelines can diverge dramatically from women’s needs and preferences (**Box 1**).

Box 1 **Can PNC guidelines be flexible enough to meet women’s and families’ needs?**

Although health workers often find themselves adapting PNC guidelines to the needs of women in their care, these processes are rarely documented. For example, when implementing the Baby Friendly Hospital Initiative in Norway, 24-hour rooming-in was considered a standard practice to support skin-to-skin contact and breastfeeding. After listening to women, it was decided the benefits of rooming-in should be explained but not made mandatory, and women who wanted to rest without their babies were offered the option of having their baby looked after by staff members for a few hours in the nursery [42]. Studies identified in this review show that women-centered approaches, promoting individualised and tailored PNC for women are possible [43]. However, such approaches need to grapple with challenges posed by the busy nature of postnatal wards. Hospital routines, even when promoting parent-infant closeness, can cause separation in the birthing unit even when PNC guidelines are being implemented as intended, for example during routine newborn checks [44]. This adaptiveness can be maintained after discharge, for example a project in France offered “home visit support to families presenting demographic characteristics associated with a higher incidence of subsequent maternal postpartum depression and infant mental health problems” [45] An assessment of this intervention showed that while home visitors were frustrated by not always being able to meet their own visit goals, the flexibility to adapt to families’ circumstances was appreciated, for example by having time to discuss administrative difficulties encountered by families before discussing the health issues targeted by the intervention. The authors therefore recommended that home visitors “adapt their curriculum to the reality and the social, psychological and health needs of the targeted population to ensure that the intervention has an impact on the individual and the community”. These adaptive approaches raise questions about the role of standard guidelines to promote consistency of high-quality care for all, and to what extent they can or should support the process of context- and woman-specific adaptation.

#### Theme 2. Navigating PNC across provision platforms

A dimension disrupting PNC provision in general and the implementation of PNC guidelines in particular is the absence of a single provision platform to offer, access and document provision of services, due to the nature of PNC being spread across health and social service providers, within a global trend toward shorter lengths of stay in health facilities after birth [46]. This “increases demands on community postnatal services, the quantity and quality of which appears to vary globally” [47]. The two main settings within which PNC is offered are health facilities (on an in-patient or out-patient basis) or in the community, meaning that continuity of care is a key challenge for PNC [48]. Fragmentation means that there are missed opportunities for women and newborns to receive comprehensive care as a unit, even when they seek care for one of the individuals in the dyad [49]. Navigating the complexity of PNC services requires understanding the system and having resources to access and negotiate services. Some interventions identified in this review address these broader structural inequalities and power asymmetries in relation to PNC use. For example, studies presented interventions to improve care in indigenous primary health centers in Australia [50], programmes and providers’ networks to help poorer and more marginalised women navigate, organise and use postnatal services [51,52], and an intervention to support perinatal mental health for women of refugee background having increased vulnerability due to pre- and post-settlement stressors and encountering barriers to access services such as knowledge of the health system and language barriers (**Box 2**) [53].

Box 2 **Identifying and overcoming intersectional inequities in fragmented service environment**

In some settings, PNC after discharge from the birth facility needs to be arranged by families themselves, putting some families who may not have the required knowledge and resources to navigate such services at a disadvantage. Grylka-Baeschlin and colleagues [52] have assessed if a network of midwives in Switzerland called “Familystart Zurich” allowing women to be connected to providers was reaching women who would otherwise encounter barriers to access postpartum care. This study showed that the network was successful in facilitating access to care for women struggling to organise postpartum care by themselves. In Chicago (USA), a postpartum programme called “Navigating New Motherhood”, which was developed to improve use of postnatal services among low-income women, hired a ‘patient navigator’ to address barriers to postpartum care [51]. The “navigator” was responsible for supportive and logistic responsibilities for enrolled patients between giving birth and postpartum visit completion. The programme was associated with improved retention in postpartum care. Both examples highlight the complexities of self-organising PNC for women after hospital discharge, especially for certain groups of women, and offer insights into providing social support for them.

#### Theme 3. PNC as a connecting and “teachable” moment within the life-course

The third theme is around the postnatal period as an important moment to focus attention to avoid mortality and morbidity and improve long-term outcomes for women and newborns (**Box 3**). It is presented as a teachable moment to provide information to women on a wide range of issues, ranging from breastfeeding, family planning, lifestyle factors, and postpartum depression, to continuity of care for complications (e.g. diabetes diagnosed in pregnancy). PNC also offers a window of opportunity to connect women to other health services. This results in efforts to deliver interventions within the relatively short window of time during which contact with the woman, newborn and family is in place postnatally. It also increases the number of care elements to be provided within outpatient consultations or postnatal visits. This puts pressure on health workers, who might not have the knowledge, skills or confidence to provide such additional care elements; and on the women, who might rather prefer to discuss other individual needs during this interaction. Some studies in this review recommend distributing some of the diagnostic and educational components from postnatal to antenatal care, where it might be more easily integrated, delivered, and digested [55].

Box 3 **The “teachable moment” in the postpartum context**

The information content shared with women in the postnatal period exceeds information directly related to their and their newborn’s care and needs in the short term. A first-time mother in Switzerland related, “I felt extremely exhausted... And then, on the first day, one gets thousands of pieces of information - how one must look after the child now and this and that, and the midwife really told me a lot, she did a lot and explained a lot to me, but I was so exhausted, absolutely empty. I could not take it at all in properly and I couldn't perceive the child properly either” [54]. In the UK, General Practitioners (GPs) have mixed perceptions on whether behavior change should be part of the six-week postnatal check. One GP observed that “They’re (mothers) often totally worn out, they’re often not happy with the way they look ‘cause they’re often overweight, they’ve got stitching. Yeah I think it’s probably not the best moment to suggest major lifestyle changes” [55]. In Tanzania, nurses reported that physical pain prevented postpartum women in hospitals from concentrating on the information content, while the low ratio of nurses to midwives meant that health workers may have to prioritise clinical tasks over patient education [56]. The perinatal period is also recognised as an opportunity to screen for women suffering from depression, and in Zimbabwe for example, “the postnatal period can also be used to screen for those women who may have missed screening or screened negative at the ANC stage”. This screening opportunity could be impeded by challenges related to low routine PNC uptake [57]. Studies from Zimbabwe suggest exploring connections with traditional medical practitioners to raise awareness on depression and potentially refer women who they identify as at risk of postnatal depression to the adequate level of care [57].

## DISCUSSION

This scoping review and critical interpretive synthesis of 43 studies identified several facilitators and barriers to implementing routine guidelines in provision of PNC to women. From the health care provision perspective, the barriers start at the higher level with the lack of prioritisation of PNC at the health system/policy/financing level and lack of PNC guideline adaptation. These high-level barriers materialise at the levels of a health care facility, team and health worker as barriers related to staff shortages, heavy workloads, infrastructural barriers to PNC provision, and lack of time to cover all PNC elements in one visit. The misalignment of standardised guidelines and practices to women’s individual postpartum needs, and the fragmentation of care between various health workers, facilities, platforms and services, were the overarching challenges to how women perceive, interact with, and seek PNC. The review documents several examples of facilitators that contribute to successfully overcoming many of these implementation challenges. Nonetheless, facilitators at the health system and policy level were not explicitly studied or mentioned in the existing literature.

We applied a critical global health lens to synthesise new constructs in the literature including: finding a balance between standardisation and individualisation of PNC, the fragmented PNC provision landscape complicating the experiences of women with intersecting vulnerabilities, and the heavy reliance on the short postpartum period as an opportunity to educate and retain women and newborns in the health system. Relying on the interpretive synthesis method is one of the strengths of this review, allowing us to identify specific topics as well as overarching themes in the literature following a rigorous screening and data extraction process. Additionally, our team involved researchers and practitioners from multiple disciplinary backgrounds, bringing varied perspectives to the interpretation of the results. We consider this multi-disciplinarity as an asset, as it challenged some of our pre-existing assumptions iteratively and allowed us to build a multifaced critique on facilitators and barriers to PNC (for example in building categories to label models of care, or how we are approaching certain definitions in a flexible manner; or to challenge normative assumptions on what PNC should look like in terms of postnatal visits, content of care etc).

We identified gaps in the literature on PNC guideline implementation from LMIC settings, as well as lack of studies on guidelines of PNC for adolescents and women who gave birth by cesarean section, studies looking at varied approaches to PNC provision including group postnatal care [58], peer support groups [59] and self-care [60], studies looking at PNC provision by sectors (public, private-for-profit, private not-for-profit), studies from humanitarian settings and marginalised populations including those in very remote areas, conflict settings and very poor populations. The largest proportion of included studies assessed routine perinatal mental health care as the main element of PNC, with few studies looking at the provision of preventive elements of PNC such as assessment of and counselling on identification of danger-signs.

The Dixon-Woods approach [33] to critical interpretive synthesis involved both induction and interpretation, leading us to question the framing of widely-adopted concepts and terms employed in PNC guidelines and models, such as routine care. We question the meaning of “routine postnatal care” from two perspectives. First, describing care as routine refers to a habit, a repetitive event, a customary or integral part of life. While this description applies to those who provide the care (which is a daily habit to health workers), it might not be perceived in the same way by women who are the intended recipients and expected users of the services. Depicting PNC elements as routine fails to take into consideration variabilities in care-seeking behaviors among postpartum women from various cultures, health care systems, socio-economic classes [61], migration status, and other structural determinants of health. Other studies have explored how labeling care as routine may normalise some elements of care while neglecting obstacles encountered by some to seek such health services [62,63]. For example, a recent study in Nigeria showed that one of the main reasons why women do not seek postnatal care for themselves is because they report that they are healthy [64]. This implies a health-seeking behavior where women perceive a lack of need for routine care because of the absence of illness. Interactions with the health care system are not perceived as routine parts of women’s daily lives and in many situations present interruptions to women’s daily routines, considering the amount of time, effort, and sometime cost that goes into understanding when, where and who provides PNC, organising the appointments, and seeking care, which is not always a straightforward system to maneuver [65]. Adopting a woman-centered PNC approach, which is to understand physical and/or psychological co-morbidities that are not specific to maternity but require care after/between pregnancies, requires a deeper understanding of women’s varied health care seeking behaviors [66]. Acknowledging the complexity involved in PNC seeking and use is a first step to understanding women’s trajectories in the care system, and a pre-requisite to designing systems that serve women, instead of systems that add-on to their responsibilities and therefore create a barrier to implementation by design.

Second, standardisation of routine PNC provision to all women, particularly through a process of translation of guidelines into the form of checklists, are challenging to implement precisely because they fail to meet the needs and preferences of all women [25]. While this standardisation is important from a clinical standpoint, it fails to consider the social determinants of health which predispose them to financial difficulties, stigma, and discrimination when receiving PNC. Routinisation or standardisation of care additionally reduces women’s involvement in decision-making about the care they receive [67]. Adapting the guideline to the need and timing of the woman involves checking women’s preferences about the elements of care they need and focusing on a more holistic well-being approach to care rather than a checklist approach [68,69]. It may involve initiating conversations during pregnancy and antenatal care visits about what to expect for PNC [67], involving partners and families in pre-discharge discussions and counselling [70], integrating or combining clinical and traditional care models that prioritise rest and sleep for the woman, and/or allowing for flexibility in the schedule of PNC visits to be able to see women at times/intervals that suit them and cater for their needs [24]. Individualised person-centered care fosters a more equitable approach to care provision, and can effectively and efficiently target those most likely to benefit from the care, while reducing the risk of leaving anyone behind [24,71].

Our study shows that one of the main challenges of routine PNC guideline implementation is lack of adaptability to the context in terms of infrastructural limitations and human resource availability [72]. In many contexts, basic resources and infrastructure are suboptimal for the provision of even basic PNC elements [73]. A recent study conducted in five low- and middle-income countries revealed that none of the health care facilities could provide all essential components of postnatal care [74]. A more favorable policy environment for maternal health, measured in terms of national supportive structures and standards, service access policies, clinical guidelines, and reporting and review systems, is associated with higher coverage of maternal health services including for postnatal care in LMICs [75]. The mechanisms through which the policy environment can influence care use are complex, and not well studied, yet political commitment could be addressing issues related to resource limitations, by increasing financing and support to the sector. Nonetheless, such complex changes require long-term investments to address gaps in physical infrastructure and human resources. A short-term solution to this challenge in managing low-resource availability is being suggested as tailoring guidelines to match the resources rather than aiming to fulfil too much in resource-limited settings, which can be extremely de-motivating for health workers [72]. It would be expected for guidelines to evolve as appropriate resources are made available. PNC guideline co-design with end-users including health workers and postpartum women can contribute to ensuring feasibility, adaptability, and acceptability in various settings.

The modalities of PNC provision documented in the literature involve multiple transitions between different health workers, facilities, settings (care facilities vs community/home), communication methods (e.g. digital platform for information sharing), sectors (health care and social services) [66], all within a very short time period when women are already going through a life-changing transition. The fragmentation of care and support means that without inter-services communication and coordination, women face challenges to access information and services, delay or forgo health care, and receive conflicting information from a diverse range of professionals [65,66], or that the source of information is inadequate or not favored by women [76]. The gap in coordination and leadership over the postnatal periods makes it more difficult for stakeholders (including health workers, women and families) to navigate this fragmentation [66]. The multitude of platforms for support availability in PNC also complicates the pathways that PNC providers should navigate, with little or unclear guidance on how the systems are interlinked, which blocks the continuity of care for women. For example, screening for certain conditions is not coupled with adequate referral to specialist care if a problem is detected, such as the case for perinatal mental health screening [77]. In other situations, guidelines are designed in a way to foster linkage between PNC for the woman and the newborn [29], however, infrastructural limitations could hinder this linkage. The way that services for the woman and newborn (such as for newborn vaccination) are organised ensures that the timing of the visits for both is synchronised in order to reduce the number of visits and the burden of travel to the health facility [29]. While this model is beneficial for women, the physical structure of health facilities does not always enable this linkage by having outpatient PNC clinics for women located far from the vaccination unit. In some high-income countries, grassroots community groups are being established by mothers - for mothers, with a focus on perinatal mental health and social support, mostly offered online for ease and convenience to new mothers [78-80]. While these programmes have potential to reduce the risk of postpartum depression [81], they have not been formally evaluated in terms of impact and equity in reach.

From the provision perspective, it is important to consider the working environment of health workers who face particular systematic challenges of working in understaffed and crowded facilities as barriers to guideline implementation [73]. In such circumstances, it can be difficult to explore individual needs of women and tailor the content of care considering the lack of additional structural support and training [68,69], especially that health workers perceive that they lack access to clear and detailed guidance for the postnatal discharge process [61]. Nonetheless, our review points to existing practices implemented by health workers to adapt guidelines to contexts and to respond to women’s concerns, and valuable lessons can be drawn from these experiences. Some practices are simple yet essential for building rapport with women and making them more comfortable during the PNC consultation. As do some GPs in the UK by starting the check with congratulating them on their baby and listening to their concerns, thus creating a relation with women and providing a consultation (or part of it) led by women [55]. Health workers’ agency in decision-making, as well as their access to evidence and knowledge, prove important for them initiating some adaptions to guidelines, as was the case in Norway regarding recommendations on timing of pacifier avoidance [42]. For informing higher-level (e.g. health district or national level) adaptations, having a platform that is receptive to health workers’ suggestions is essential to support them in transforming their ideas into implemented adaptations. In Tanzania, health workers suggested initiatives for overcoming existing inconsistencies in record keeping, thus contributing to improving the health information system for postnatal care [82].

### Limitations

Despite its strengths, this work also has some limitations. Our scope and strategy did not capture initiatives that were not directly related to guideline implementation, meaning that studies focusing only on women’s perspectives and peer network initiatives were excluded. This limits our understanding of experiences of care and navigating postnatal services from users’ perspectives. Additionally, some papers were excluded from core thematic analyses because they did not inform specifically on facilitators and barriers of PNC but deemed relevant for background information and conceptualisation. The search was conducted in 2021, meaning that this review summarises the evidence prior to the COVID-19 pandemic, which has brought to the light numerous new challenges to PNC provision and guideline implementation [83]. We also acknowledge that the focus of this study on PNC for women, which was necessary in order to manage its scope and breadth, might omit some evidence relevant specifically to implementation of routine PNC guidelines for newborns. However, many of the findings and themes incorporated in our findings relate specifically to the woman-baby dyad.

## CONCLUSIONS

This interpretive synthesis of evidence documented how the fragmented nature of PNC provision, a large part of which requires social support and services, presents specific challenges to developing, adapting, and implementing routine PNC guidelines. Additionally, a gap exists in the evidence on simplifying the complex PNC provision landscape to make it more accessible and equitable for women with various intersecting inequalities and vulnerabilities. Finally, this review found a gap in the literature regarding how processes of individualising routine PNC guidelines and care provision can be applied in practice and understand how the health care system can support health workers in providing woman-centered PNC across a range of global settings.

## Additional material


Online Supplementary Document

## References

[1] World Health Organization. Trends in maternal mortality 2000 to 2020: estimates by WHO, UNICEF, UNFPA, World Bank Group and UNDESA/Population Division. Geneva; 2023.

[2] UNICEF, WHO, World Bank Group, the United Nations Population Division. A Neglected Tragedy - The global burden of stillbirths. 2020.

[3] Global strategy for women's, children's and adolescents' health (2016-2030). Available: https://platform.who.int/data/maternal-newborn-child-adolescent-ageing/global-strategy-data. Accessed: 18 August 2023.

[4] Sustainable development goal 3: Ensure healthy lives and promote well-being for all at all ages. Available: https://sustainabledevelopment.un.org/sdg3. Accessed: 22 September 2023.

[5] World Health Organization. SDG Target 3.1: Reduce the global maternal mortality ratio to less than 70 per 100,000 live births. Available: https://www.who.int/data/gho/data/themes/topics/topic-details/GHO/sdgtarget3-1-reduce-maternal-mortality. Accessed: 1 September 2023.

[6] Campbell OMR Calvert C Testa A Strehlow M Benova L Keyes E The scale, scope, coverage, and capability of childbirth care. Lancet. 2016;388:2193-208. 10.1016/S0140-6736(16)31528-8 27642023

[7] United Nations Inter-agency Group for Child Mortality Estimation. Levels & Trends in Child Mortality - Report 2022.Available: https://childmortality.org/wp-content/uploads/2023/01/UN-IGME-Child-Mortality-Report-2022.pdf. Accessed: 8 September 2023.

[8] Kruk ME Gage AD Arsenault C Jordan K Leslie HH Roder-DeWan S High-quality health systems in the Sustainable Development Goals era: time for a revolution. Lancet Glob Health. 2018;6:e1196-252. 10.1016/S2214-109X(18)30386-3 30196093 PMC7734391

[9] Lawn JE Blencowe H Oza S You D Lee AC Waiswa P Every Newborn: progress, priorities, and potential beyond survival. Lancet. 2014;384:189-205. 10.1016/S0140-6736(14)60496-7 24853593

[10] Ronsmans C Graham WJ Maternal mortality: who, when, where, and why. Lancet. 2006;368:1189-200. 10.1016/S0140-6736(06)69380-X 17011946

[11] Merdad L Ali MM Timing of maternal death: Levels, trends, and ecological correlates using sibling data from 34 sub-Saharan African countries. PLoS One. 2018;13:e0189416. 10.1371/journal.pone.0189416 29342157 PMC5771557

[12] Alliance for Maternal and Newborn Health Improvement (AMANHI) mortality study group Population-based rates, timing, and causes of maternal deaths, stillbirths, and neonatal deaths in south Asia and sub-Saharan Africa: a multi-country prospective cohort study. Lancet Glob Health. 2018;6:e1297-308. 10.1016/S2214-109X(18)30385-1 30361107 PMC6227247

[13] Fort AL Coverage of post-partum and post-natal care in Egypt in 2005–2008 and Bangladesh in 2004–2007: levels, trends and unmet need. Reprod Health Matters. 2012;20:81-92. 10.1016/S0968-8080(12)39600-6 22789085

[14] Gazeley U Reniers G Eilerts-Spinelli H Prieto JR Jasseh M Khagayi S Women’s risk of death beyond 42 days post partum: a pooled analysis of longitudinal Health and Demographic Surveillance System data in sub-Saharan Africa. Lancet Glob Health. 2022;10:e1582-9. 10.1016/S2214-109X(22)00339-4 36240825

[15] Tinker A ten Hoope-Bender P Azfar S Bustreo F Bell R A continuum of care to save newborn lives. Lancet. 2005;365:822-5. 10.1016/S0140-6736(05)71016-3 15752509

[16] Sacks E Masvawure TB Atuyambe LM Neema S Macwan’gi M Simbaya J Postnatal care experiences and barriers to care utilization for home-and facility-delivered newborns in Uganda and Zambia. Matern Child Health J. 2017;21:599-606. 10.1007/s10995-016-2144-4 27475823

[17] Countdown to 2030 Collaboration Countdown to 2030: tracking progress towards universal coverage for reproductive, maternal, newborn, and child health. Lancet. 2018;391:1538-48. 10.1016/S0140-6736(18)30104-1 29395268

[18] Benova L Owolabi O Radovich E Wong KLM Macleod D Langlois EV Provision of postpartum care to women giving birth in health facilities in sub-Saharan Africa: A cross-sectional study using Demographic and Health Survey data from 33 countries. PLoS Med. 2019;16:e1002943. 10.1371/journal.pmed.1002943 31644531 PMC6808422

[19] Sacks E Langlois ÉV Postnatal care: increasing coverage, equity, and quality. Lancet Glob Health. 2016;4:e442-3. 10.1016/S2214-109X(16)30092-4 27185467

[20] Mi T Hung P Li X McGregor A He J Zhou J Racial and ethnic disparities in postpartum care in the greater Boston area during the COVID-19 pandemic. JAMA Network Open. 2022;5:e2216355. 10.1001/jamanetworkopen.2022.16355 35737390 PMC9226999

[21] Langlois ÉV Miszkurka M Zunzunegui MV Ghaffar A Ziegler D Karp I Inequities in postnatal care in low-and middle-income countries: a systematic review and meta-analysis. Bull World Health Organ. 2015;93:259-70G. 10.2471/BLT.14.140996 26229190 PMC4431556

[22] Madaj B Gopalakrishnan S Quach A Filiaci S Traore A Bakusa D Where is the ‘C’in antenatal care and postnatal care: A multi-country survey of availability of antenatal and postnatal care in low-and middle-income settings. BJOG. 2022;129:1546-57. 10.1111/1471-0528.17106 35106907 PMC9541911

[23] Gresh A Cohen M Anderson J Glass N Postpartum care content and delivery throughout the African continent: An integrative review. Midwifery. 2021;97:102976. 10.1016/j.midw.2021.102976 33740519 PMC8985233

[24] Sacks E Finlayson K Brizuela V Crossland N Ziegler D Sauvé C Factors that influence uptake of routine postnatal care: Findings on women’s perspectives from a qualitative evidence synthesis. PLoS One. 2022;17:e0270264. 10.1371/journal.pone.0270264 35960752 PMC9374256

[25] Javadi D Sacks E Brizuela V Finlayson K Crossland N Langlois EV Factors that influence the uptake of postnatal care among adolescent girls: a qualitative evidence synthesis. BMJ Glob Health. 2023;8 Suppl 2:e011560. 10.1136/bmjgh-2022-011560 37137533 PMC10163540

[26] Finlayson K Sacks E Brizuela V Crossland N Cordey S Ziegler D Factors that influence the uptake of postnatal care from the perspective of fathers, partners and other family members: a qualitative evidence synthesis. BMJ Glob Health. 2023;8 Suppl 2:e011086. 10.1136/bmjgh-2022-011086 37137532 PMC10163465

[27] Sacks E Kinney MV Respectful maternal and newborn care: building a common agenda. Reprod Health. 2015;12:46. 10.1186/s12978-015-0042-7 25986552 PMC4460639

[28] Negero MG Sibbritt D Dawson A Women’s utilisation of quality antenatal care, intrapartum care and postnatal care services in Ethiopia: a population-based study using the demographic and health survey data. BMC Public Health. 2023;23:1174. 10.1186/s12889-023-15938-8 37337146 PMC10278283

[29] World Health Organization. WHO recommendations on maternal and newborn care for a positive postnatal experience. Geneva: World Health Organization; 2022 2022. 35467813

[30] Wojcieszek AM Bonet M Portela A Althabe F Bahl R Chowdhary N WHO recommendations on maternal and newborn care for a positive postnatal experience: strengthening the maternal and newborn care continuum. BMJ Glob Health. 2023;8 Suppl 2:e010992. 10.1136/bmjgh-2022-010992 36717156 PMC9887708

[31] Arksey H O’Malley L Scoping studies: towards a methodological framework. Int J Soc Res Methodol. 2005;8:19-32. 10.1080/1364557032000119616

[32] Tricco AC Lillie E Zarin W O’Brien KK Colquhoun H Levac D PRISMA extension for scoping reviews (PRISMA-ScR): checklist and explanation. Ann Intern Med. 2018;169:467-73. 10.7326/M18-0850 30178033

[33] Dixon-Woods M Cavers D Agarwal S Annandale E Arthur A Harvey J Conducting a critical interpretive synthesis of the literature on access to healthcare by vulnerable groups. BMC Med Res Methodol. 2006;6:35. 10.1186/1471-2288-6-35 16872487 PMC1559637

[34] Mothupi MC Knight L Tabana H Measurement approaches in continuum of care for maternal health: a critical interpretive synthesis of evidence from LMICs and its implications for the South African context. BMC Health Serv Res. 2018;18:539. 10.1186/s12913-018-3278-4 29996924 PMC6042348

[35] Benova L, Semaan A. Facilitators and barriers to postnatal care guideline implementation: A systematic scoping review. Available: https://www.protocols.io/view/facilitators-and-barriers-to-postnatal-care-guidel-j8nlk4m76g5r/v1. Accessed: 24 March 2021.

[36] Munabi-Babigumira S Lewin S Glenton C Velez M Gonçalves-Bradley DC Bohren MA Factors that influence the provision of postnatal care by health workers: a qualitative evidence synthesis. Cochrane Database Syst Rev. 2021;2021:CD014790. 10.1002/14651858.CD011558.pub2 PMC5721625 29148566

[37] Bramer WM Giustini D de Jonge GB Holland L Bekhuis T De-duplication of database search results for systematic reviews in EndNote. J Med Libr Assoc. 2016;104:240. 10.3163/1536-5050.104.3.014 27366130 PMC4915647

[38] Bamuya C Correia JC Brady EM Beran D Harrington D Damasceno A Use of the socio-ecological model to explore factors that influence the implementation of a diabetes structured education programme (EXTEND project) in Lilongwe, Malawi and Maputo, Mozambique: a qualitative study. BMC Public Health. 2021;21:1355. 10.1186/s12889-021-11338-y 34238258 PMC8268266

[39] Adams V What is critical global health? Med Anthropol Theory. 2016;3:186.

[40] Koon AD Mendenhall E Hawkins B Framing: realising the potential of a contested concept. Lancet. 2022;400:561. 10.1016/S0140-6736(22)01484-2 35988565

[41] Fedock GL Alvarez C Differences in Screening and Treatment for Antepartum Versus Postpartum Patients: Are Providers Implementing the Guidelines of Care for Perinatal Depression? J Womens Health (Larchmt). 2018;27:1104-13. 10.1089/jwh.2017.6765 29757074

[42] Hansen MN Bærug A Nylander G Häggkvist AP Tufte E Alquist R Challenges and successes: the Baby-Friendly Initiative in Norway. J Hum Lact. 2012;28:285-8. 10.1177/0890334412444162 22723529

[43] Yelland J McLachlan H Forster D Rayner J Lumley J How is maternal psychosocial health assessed and promoted in the early postnatal period? Findings from a review of hospital postnatal care in Victoria, Australia. Midwifery. 2007;23:287-97. 10.1016/j.midw.2006.06.003 17116348

[44] Niela-Vilén H Feeley N Axelin A Hospital routines promote parent-infant closeness and cause separation in the birthing unit in the first 2 hours after birth: A pilot study. Birth. 2017;44:167-72. 10.1111/birt.12279 28198043

[45] Saïas T Lerner E Greacen T Simon-Vernier E Emer A Pintaux E Evaluating fidelity in home-visiting programs a qualitative analysis of 1058 home visit case notes from 105 families. PLoS One. 2012;7:e36915. 10.1371/journal.pone.0036915 22629341 PMC3356353

[46] Clark HD Keely E Getting mothers with gestational diabetes to return for postpartum testing: What works and what does not. Diabetes Manage. 2012;2:33-39. 10.2217/dmt.11.64

[47] Cegolon L Mastrangelo G Maso G Pozzo GD Heymann WC Ronfani L Determinants of length of stay after cesarean sections in the Friuli Venezia Giulia Region (North-Eastern Italy), 2005-2015. Sci Rep. 2020;10:19238. 10.1038/s41598-020-74161-2 33159096 PMC7648096

[48] Forster DA McKay H Powell R Wahlstedt E Farrell T Ford R The structure and organisation of home-based postnatal care in public hospitals in Victoria, Australia: A cross-sectional survey. Women Birth. 2016;29:172-9. 10.1016/j.wombi.2015.10.002 26563639

[49] Haskins LJ Phakathi SP Grant M Mntambo N Wilford A Horwood CM Fragmentation of maternal, child and HIV services: A missed opportunity to provide comprehensive care. Afr J Prim Health Care Fam Med. 2016;8:e1-8. 10.4102/phcfm.v8i1.1240 28155320 PMC5153411

[50] Rumbold AR Bailie RS Si D Dowden MC Kennedy CM Cox RJ Assessing the quality of maternal health care in Indigenous primary care services. Med J Aust. 2010;192:597-8. 10.5694/j.1326-5377.2010.tb03646.x 20477738

[51] Yee LM Martinez NG Nguyen AT Hajjar N Chen MJ Simon MA Using a Patient Navigator to Improve Postpartum Care in an Urban Women’s Health Clinic. Obstet Gynecol. 2017;129:925-33. 10.1097/AOG.0000000000001977 28383374 PMC5400713

[52] Grylka-Baeschlin S Iglesias C Erdin R Pehlke-Milde J Evaluation of a midwifery network to guarantee outpatient postpartum care: a mixed methods study. BMC Health Serv Res. 2020;20:565. 10.1186/s12913-020-05359-3 32571320 PMC7310082

[53] Nithianandan N Gibson-Helm M McBride J Binny A Gray KM East C Factors affecting implementation of perinatal mental health screening in women of refugee background. Implement Sci. 2016;11:150. 10.1186/s13012-016-0515-2 27863498 PMC5116191

[54] Kurth E Spichiger E Zemp Stutz E Biedermann J Hösli I Kennedy HP Crying babies, tired mothers - challenges of the postnatal hospital stay: an interpretive phenomenological study. BMC Pregnancy Childbirth. 2010;10:21. 10.1186/1471-2393-10-21 20462462 PMC2879231

[55] Talbot H Strong E Peters S Smith DM Behaviour change opportunities at mother and baby checks in primary care: a qualitative investigation of the experiences of GPs. Br J Gen Pract. 2018;68:e252-9. 10.3399/bjgp18X695477 29530920 PMC5863679

[56] Dol J Kohi T Campbell-Yeo M Tomblin Murphy G Aston M Mselle L Exploring maternal postnatal newborn care postnatal discharge education in Dar es Salaam, Tanzania: Barriers, facilitators and opportunities. Midwifery. 2019;77:137-43. 10.1016/j.midw.2019.07.009 31325744

[57] January J Chimbari MJ Opportunities and obstacles to screening for perinatal depression among women in Zimbabwe: A narrative review of literature. S Afr J Psychiatr. 2018;24:1127. 10.4102/sajpsychiatry.v24i0.1127 30263213 PMC6138181

[58] Martens N Crone MR Hindori-Mohangoo A Hindori M Reis R Hoxha IS Group Care in the first 1000 days: implementation and process evaluation of contextually adapted antenatal and postnatal group care targeting diverse vulnerable populations in high-, middle- and low-resource settings. Implement Sci Commun. 2022;3:125. 10.1186/s43058-022-00370-7 36424641 PMC9694875

[59] Centering Healthcare InstituteAvailable: https://centeringhealthcare.org/. Accessed: 15 May 2023.

[60] Nguyen P-Y Caddy C Wilson AN Blackburn K Page MJ Gülmezoglu AM Self-care interventions for preconception, antenatal, intrapartum and postpartum care: a scoping review. BMJ Open. 2023;13:e068713. 10.1136/bmjopen-2022-068713 37164476 PMC10173967

[61] Harvey CM Smith H Portela A Movsisyan A Stakeholder’s perspectives of postnatal discharge: a qualitative evidence synthesis. BMJ Glob Health. 2023;8 Suppl 2:e011766. 10.1136/bmjgh-2023-011766 37553175 PMC10414110

[62] Green CA Johnson KM Yarborough BJ Seeking, delaying, and avoiding routine health care services: patient perspectives. Am J Health Promot. 2014;28:286-93. 10.4278/ajhp.120702-QUAL-318 23971522 PMC3933475

[63] Ceasar JN Ayers C Andrews MR Claudel SE Tamura K Das S Unfavorable perceived neighborhood environment associates with less routine healthcare utilization: Data from the Dallas Heart Study. PLoS One. 2020;15:e0230041. 10.1371/journal.pone.0230041 32163470 PMC7067436

[64] Onwuka CI Ezugwu EC Obi SN Onwuka C Dim CC Chigbu C Postnatal care services use by mothers: A comparative study of defaulters versus attendees of postnatal clinics in Enugu. PLoS One. 2023;18:e0280315. 10.1371/journal.pone.0280315 36996250 PMC10062588

[65] Tully KP Stuebe AM Foreword: Respectful, Equitable, and Supportive Postpartum Care. Clin Obstet Gynecol. 2022;65:538-49. 10.1097/GRF.0000000000000734 35797646 PMC9339469

[66] Shakespeare J Duff E Bick D How many more reviews are needed? Joined-up policy and investment in England’s maternity units is needed now to improve postnatal and inter-pregnancy care. Midwifery. 2022;113:103469. 10.1016/j.midw.2022.103469 36045018

[67] Harris KA Etienne S Arrington LA Postnatal Unit Care and Safe Transition Home. Clin Obstet Gynecol. 2022;65:563-76. 10.1097/GRF.0000000000000732 35797543

[68] Royal College of Midwives. Supporting women seeking care outside guidance. Available: from: https://www.rcm.org.uk/news-views/rcm-opinion/2022/supporting-women-seeking-care-outside-guidance/. Accessed 12 August 2023.

[69] Ansmann L Pfaff H Providers and Patients Caught Between Standardization and Individualization: Individualized Standardization as a Solution; Comment on “(Re) Making the Procrustean Bed? Standardization and Customization as Competing Logics in Healthcare”. Int J Health Policy Manag. 2018;7:349-52. 10.15171/ijhpm.2017.95 29626403 PMC5949226

[70] Murthy S Yan SD Alam S Kumar A Rangarajan A Sawant M Improving neonatal health with family-centered, early postnatal care: A quasi-experimental study in India. PLOS Glob Public Health. 2023;3:e0001240. 10.1371/journal.pgph.0001240 37228043 PMC10212134

[71] Muriuki A Yahner M Kiragu M Graft-Johnson Jd, Izulla P. On the road to universal coverage of postnatal care: considerations for a targeted postnatal care approach for at-risk mother–baby dyads in low-income and middle-income countries informed by a consultation with global experts. BMJ Open. 2022;12:e058408. 10.1136/bmjopen-2021-058408 35701048 PMC9198691

[72] Maaløe N Ørtved AMR Sørensen JB Sequeira Dmello B van den Akker T Kujabi ML The injustice of unfit clinical practice guidelines in low-resource realities. Lancet Glob Health. 2021;9:e875-9. 10.1016/S2214-109X(21)00059-0 33765437 PMC7984859

[73] Namutebi M Nalwadda GK Kasasa S Muwanguzi PA Kaye DK Midwives’ perceptions towards the ministry of health guidelines for the provision of immediate postpartum care in rural health facilities in Uganda. BMC Pregnancy Childbirth. 2023;23:261. 10.1186/s12884-023-05585-7 37072738 PMC10111670

[74] Madaj B Gopalakrishnan S Quach A Filiaci S Traore A Bakusa D Where is the ‘C’ in antenatal care and postnatal care: A multi-country survey of availability of antenatal and postnatal care in low- and middle-income settings. BJOG. 2022;129:1546-57. 10.1111/1471-0528.17106 35106907 PMC9541911

[75] Creanga AA Dohlsten MA Stierman EK Moran AC Mary M Katwan E Maternal health policy environment and the relationship with service utilization in low- and middle-income countries. J Glob Health. 2023;13:04025. 10.7189/jogh.13.04025 36892948 PMC9997690

[76] Klingberg S Motlhatlhedi M Mabena G Mooki T Verdezoto N Densmore M “Must you make an app?” A qualitative exploration of socio-technical challenges and opportunities for designing digital maternal and child health solutions in Soweto, South Africa. PLOS Glob Public Health. 2022;2:e0001280. 10.1371/journal.pgph.0001280 36962834 PMC10021787

[77] Viveiros CJ Darling EK Perceptions of barriers to accessing perinatal mental health care in midwifery: A scoping review. Midwifery. 2019;70:106-18. 10.1016/j.midw.2018.11.011 30611114

[78] Mothers talking. Available: https://www.naomistadlen.com/mothers-talking/. Accessed: 1 September 2023.

[79] The motherhood group. Available: https://themotherhoodgroup.org. Accessed: 1 September 2023.

[80] Postpartum support international. Available: https://www.postpartum.net/. Accessed: 1 September 2023.

[81] Shorey S Chee CYI Ng ED Lau Y Dennis C-L Chan YH Evaluation of a Technology-Based Peer-Support Intervention Program for Preventing Postnatal Depression (Part 1): Randomized Controlled Trial. J Med Internet Res. 2019;21:e12410. 10.2196/12410 31469084 PMC6744221

[82] Pallangyo E Mbekenga C Olsson P Eriksson L Bergström A Implementation of a facilitation intervention to improve postpartum care in a low-resource suburb of Dar es Salaam, Tanzania. Implement Sci. 2018;13:102. 10.1186/s13012-018-0794-x 30055638 PMC6064049

[83] Semaan A Dey T Kikula A Asefa A Delvaux T Langlois EV “Separated during the first hours”—Postnatal care for women and newborns during the COVID-19 pandemic: A mixed-methods cross-sectional study from a global online survey of maternal and newborn healthcare providers. PLOS Glob Public Health. 2022;2:e0000214. 10.1371/journal.pgph.0000214 36962168 PMC10022345

