# Supplementary material for: Facilitators and barriers of implementation of routine postnatal care guidelines for women: A systematic scoping review using critical interpretive synthesis
Source: J Glob Health. 2023 Nov 24;13:04176. doi: 10.7189/jogh.13.04176 (PMC10668206; doi:10.7189/jogh.13.04176)
Supplement: Online Supplementary Document [file jogh-13-04176-s001.pdf]

S1. PRISMA-ScR Checklist

SECTION	ITEM	PRISMA-ScR CHECKLIST ITEM	REPORTED ON PAGE
**TITLE**			
Title	1	Identify the report as a scoping review.	1
**ABSTRACT**			
Structured summary	2	Provide a structured summary that includes (as applicable): background, objectives, eligibility criteria, sources of evidence, charting methods, results, and conclusions that relate to the review questions and objectives.	2
**INTRODUCTION**			
Rationale	3	Describe the rationale for the review in the context of what is already known. Explain why the review questions/objectives lend themselves to a scoping review approach.	3-4
Objectives	4	Provide an explicit statement of the questions and objectives being addressed with reference to their key elements (e.g., population or participants, concepts, and context) or other relevant key elements used to conceptualize the review questions and/or objectives.	4
**METHODS**			
Protocol and registration	5	Indicate whether a review protocol exists; state if and where it can be accessed (e.g., a Web address); and if available, provide registration information, including the registration number.	4, ref 34
Eligibility criteria	6	Specify characteristics of the sources of evidence used as eligibility criteria (e.g., years considered, language, and publication status), and provide a rationale.	4
Information sources*	7	Describe all information sources in the search (e.g., databases with dates of coverage and contact with authors to identify additional sources), as well as the date the most recent search was executed.	4 and SM2
Search	8	Present the full electronic search strategy for at least 1 database, including any limits used, such that it could be repeated.	SM2
Selection of sources of evidence†	9	State the process for selecting sources of evidence (i.e., screening and eligibility) included in the scoping review.	4-5 and SM3
Data charting process‡	10	Describe the methods of charting data from the included sources of evidence (e.g., calibrated forms or forms that have been tested by the team before their use, and whether data charting was done independently or in duplicate) and any processes for obtaining and confirming data from investigators.	5
Data items	11	List and define all variables for which data were sought and any assumptions and simplifications made.	5
Critical appraisal of individual sources of evidence§	12	If done, provide a rationale for conducting a critical appraisal of included sources of evidence; describe the methods used and how this information was used in any data synthesis (if appropriate).	Not applicable
Synthesis of results	13	Describe the methods of handling and summarizing the data that were charted.	5

SECTION	ITEM	PRISMA-ScR CHECKLIST ITEM	REPORTED ON PAGE
**RESULTS**			
Selection of sources of evidence	14	Give numbers of sources of evidence screened, assessed for eligibility, and included in the review, with reasons for exclusions at each stage, ideally using a flow diagram.	6, Figure 1, and SM4
Characteristics of sources of evidence	15	For each source of evidence, present characteristics for which data were charted and provide the citations.	Table 1 and SM5
Critical appraisal within sources of evidence	16	If done, present data on critical appraisal of included sources of evidence (see item 12).	Not applicable
Results of individual sources of evidence	17	For each included source of evidence, present the relevant data that were charted that relate to the review questions and objectives.	9-11, Figure 2 and 3
Synthesis of results	18	Summarize and/or present the charting results as they relate to the review questions and objectives.	11-12
**DISCUSSION**			
Summary of evidence	19	Summarize the main results (including an overview of concepts, themes, and types of evidence available), link to the review questions and objectives, and consider the relevance to key groups.	13-14
Limitations	20	Discuss the limitations of the scoping review process.	15
Conclusions	21	Provide a general interpretation of the results with respect to the review questions and objectives, as well as potential implications and/or next steps.	15
**FUNDING**			
Funding	22	Describe sources of funding for the included sources of evidence, as well as sources of funding for the scoping review. Describe the role of the funders of the scoping review.	16

JBI = Joanna Briggs Institute; PRISMA-ScR = Preferred Reporting Items for Systematic reviews and Meta-Analyses extension for Scoping Reviews.

* Where *sources of evidence* (see second footnote) are compiled from, such as bibliographic databases, social media platforms, and Web sites.

† A more inclusive/heterogeneous term used to account for the different types of evidence or data sources (e.g., quantitative and/or qualitative research, expert opinion, and policy documents) that may be eligible in a scoping review as opposed to only studies. This is not to be confused with *information sources* (see first footnote).

‡ The frameworks by Arksey and O'Malley (6) and Levac and colleagues (7) and the JBI guidance (4, 5) refer to the process of data extraction in a scoping review as data charting.

§ The process of systematically examining research evidence to assess its validity, results, and relevance before using it to inform a decision. This term is used for items 12 and 19 instead of "risk of bias" (which is more applicable to systematic reviews of interventions) to include and acknowledge the various sources of evidence that may be used in a scoping review (e.g., quantitative and/or qualitative research, expert opinion, and policy document).

From: Tricco AC, Lillie E, Zarin W, O'Brien KK, Colquhoun H, Levac D, et al. PRISMA Extension for Scoping Reviews (PRISMAScR): Checklist and Explanation. Ann Intern Med. 2018;169:467–473. doi: 10.7326/M18-0850.

S2: Searches by database

MEDLINE

Search algorithm: 1 AND 2 AND 3 AND 4

Concept number	Concept name	Search terms
1	Implementation	implementat* OR barrier* OR facilitat* OR quality OR challenge* OR constraint* OR difficult* OR disincentive* OR incentive* OR hamper* OR hinder* OR impairm* OR impede* OR influenc* OR motivat* OR limit* OR neglect* OR obstacle* OR promot*
2	Postnatal care	post?natal OR post?partum OR new?born OR neonatal OR perinatal OR puerperium OR puerperal OR post?parturient OR post?parturition OR afterbirth
3	Guidelines	guideline* OR recommendation* OR polic* OR care OR visit* OR follow* OR management OR service* OR discharge OR provision OR check* OR length?of?stay OR standard
4	Routine care	Routine*
Additional parameters		Restriction to HUMAN studies and published since 2000

EMBASE

Search algorithm: 1 AND 2 AND 3 AND 4

Concept number	Concept name	Search terms
1	Implementation	implementat* OR barrier* OR facilitat* OR quality OR challenge* OR constraint* OR difficult* OR disincentive* OR incentive* OR hamper* OR hinder* OR impairm* OR impede* OR influenc* OR motivat* OR limit* OR neglect* OR obstacle* OR promot*
2	Postnatal care	post?natal OR post?partum OR new?born OR neonatal OR perinatal OR puerperium OR puerperal OR post?parturient OR post?parturition OR afterbirth
3	Guidelines	guideline* OR recommendation* OR polic* OR care OR visit* OR follow* OR management OR service* OR discharge OR provision OR check* OR length?of?stay OR standard
4	Routine care	Routine*
Additional parameters		Restriction to HUMAN studies and published since 2000

GLOBAL HEALTH

Search algorithm: 1 AND 2 AND 3 AND 4

Concept number	Concept name	Search terms
1	Implementation	implementat* OR barrier* OR facilitat* OR quality OR challenge* OR constraint* OR difficult* OR disincentive* OR incentive* OR hamper* OR hinder* OR impairm* OR impede* OR influenc* OR motivat* OR limit* OR neglect* OR obstacle* OR promot* OR ~~support*~~
2	Postnatal care	post?natal OR post?partum OR new?born OR neonatal OR perinatal OR puerperium OR puerperal OR post?parturient OR post?parturition OR afterbirth

3	Guidelines	guideline* OR recommendation* OR polic* OR care OR visit* OR follow* OR management OR service* OR discharge OR provision OR check* OR length?of?stay OR standard
4	Routine care	Routine*
Additional parameters		Restriction to published since 2000

CINAHL Plus – March 23, 2021

Search algorithm: 1 AND 2 AND 3 AND 4

Concept number	Concept name	Search terms
1	Implementation	implementat* OR barrier* OR facilitat* OR quality OR challenge* OR constraint* OR difficult* OR disincentive* OR incentive* OR hamper* OR hinder* OR impairm* OR impede* OR influenc* OR motivat* OR limit* OR neglect* OR obstacle* OR promot*
2	Postnatal care	post?natal OR post?partum OR new?born OR neonatal OR perinatal OR puerperium OR puerperal OR post?parturient OR post?parturition OR afterbirth
3	Guidelines	guideline* OR recommendation* OR polic* OR care OR visit* OR follow* OR management OR service* OR discharge OR provision OR check* OR length?of?stay OR standard
4	Routine care	Routine*

S3. Inclusion and exclusion criteria applied in steps 1 and 2 of screening (title and abstract thematic mapping)

Main criteria	Specific criteria for inclusion	Criteria for exclusion
**Scope**	• Refers to postnatal care guidelines – the extent to which guidelines are known, implemented, observed, measured, or evaluated.<br>• Implementation of postnatal care elements of care – e.g., papers describing interventions to improve provision, assess any changes in provision, introduction of additional/new elements of postnatal care, evaluation of results after changes made to provision of postnatal care, etc.<br>• Presents barriers and/or facilitators of providing sufficient and good quality postnatal care<br>• Focus on routine postnatal care delivery (screening, preventive, counselling/health education, support care elements), including any referral or provider linkages (e.g., from hospital birth to home or outpatient-based postnatal care)	• Postnatal care for newborns only, withouth any element of integration with mother's postnatal care<br>• Reports on clinical outcomes in the postpartum period only, without implementation considerations
**Study Population**	• Studies focused on elements of PNC provision within a health system – health facilities, districts, national level, health workers, networks of providers.<br>• Source of data includes health system (e.g., referral functioning, review of patient records, routine data), health facilities, healthcare providers, community, guidelines, guideline reviews etc.	• Sources of data on postnatal care use or provision include recipients only (women, partners, families)
**Study design**	• Observational (prospective or retrospective), health system case study, intervention, evaluation, document/policy analysis.<br>• Quantitative, qualitative or mixed methods.<br>• Primary (original research) studies, secondary data analyses, systematic reviews.	• Conceptual frameworks<br>• Study protocols<br>• Individual patient case study<br>• Indicator validation studies<br>• Guideline document/update to clinical guidelines without analysis of barriers/facilitators
**Outcome variables**	• Any outcomes related to postnatal care use and/or service provision, including completeness/continuity of care, content, quality, satisfaction	• Maternal/foetal health outcomes in pregnancy, labour and childbirth without links to postnatal care use or provision<br>• Care beyond the postnatal period (1 year after birth), child health<br>• General maternal health service access and utilisation beyond postnatal care
**Setting**	All settings globally	-
**Time period**	Published since 2000	Published before 2000
**Language**	Any	-
**Type of paper**	Peer reviewed journal article (research study), report	Opinion piece, commentary, review, letters to editors, conference abstracts and thesis documents

S4: Broad thematic areas identified in excluded records, by step

Step of screening<br>Number of records thematically mapped	Broad thematic areas covered by records excluded
Step 1:<br>Title and abstract screening<br>(n=8,418)	- Routine newborn screening and vaccination<br>- Specific newborn screening for congenital disease, hearing loss<br>- Postnatal care for small and sick newborns, practices in neonatal intensive care units including newborn pain management<br>- Baby-friendly hospitals, breastfeeding initiation without guideline implementation considerations<br>- Provision of postpartum family planning<br>- Screening and treatment for postpartum depression (mostly maternal but some also paternal), screening tools validation studies without guideline implementation considerations<br>- HIV treatment during the maternal continuum of care, postnatal care follow-up after PMTCT or vertical exposure to HIV
Step 2:<br>Excluded in in-depth title and abstract assessment and team discussion<br>(n=146)	- Breastfeeding and mental health topics in the postpartum period, without guideline implementation considerations<br>- Postnatal care for specific sub-populations of women – routine care for women with GDM or pregnancy hypertension, without guideline implementation considerations
Step 3:<br>Full-text review<br>(n=85)	- Postnatal care from women's perspective only<br>- Care beyond the postnatal period<br>- Care for newborns only<br>- Postnatal care for specific complications (not implementation of routine PNC guidelines)

S5. Table of 43 included studies, sorted by topic and year of publication

First author (year of publication), reference	Country	Study objective(s)	Study design, methods, population/source of data	Inter-vention	Topic (routine, one element, specific target group)	PNC setting	Barriers (B) and facilitators (F) identifie
**Topic: One element of routine postnatal care**							
Steele et al (2003)[1]	UK	To describe the provision of postnatal debriefing in two Health Authority regions in England, clarify the meaning of the term postnatal debriefing, and to make recommendations on the provision of postnatal debriefing.	Observational, quantitative, maternity units	No	Element of PNC: postnatal debriefing	Hospitals (public, hospital-based maternity units)	**B:** Financial resources, staffing levels and training of staff.
Buist et al (2006)[2]	Australia	To assess the acceptability of routine screening for perinatal depression.	Observational, quantitative, women and healthcare providers	Yes	Element of PNC: perinatal depression screening	Multiple: Maternity services which routinely provide postnatal care (multiple types, including GPs)	**B:** Midwives had less experience with moo disturbances than other health professiona and rated their skills and comfort as less adequate.<br><br>**F:** Training of the health professionals abou need to talk for women
Tappin et al (2006)[3]	UK	To document the roles of individual health visitors in promoting and supporting breastfeeding in the primary care setting in Glasgow and to report any relationships between these interventions	Observational, Quantitative, healthcare providers (health visitors)	No	Element of PNC: Postnatal support for breastfeeding at 10 days and 6 weeks after birth	Community/primary	**F:** Training of providers

First author (year of publication), reference	Country	Study objective(s)	Study design, methods, population/source of data	Inter-vention	Topic (routine, one element, specific target group)	PNC setting	Barriers (B) and facilitators (F) identifie
		and routine breastfeeding rates gathered on Child Health Surveillance records linked to individual health visitors.					
Yelland et al (2007)[4]	Australia	To describe how women's maternal health, particularly at a psychosocial level, is assessed and promoted during the postnatal hospital stay	Observational, mixed-methods, public hospitals and healthcare providers	No	Element of PNC: mental health screening	Hospitals (secondary)	**B:** Busy and chaotic nature of postnatal wa and the lack of flexibility in meeting individ women's needs (difficult to tailor care to ea woman given the required checking, educa and documentation that had to be underta during relatively short hospital stays). Staff constraints and external visitors added to t difficulties in providing women-centred car Low skills and confidence of midwives in providing some elements of PNC, for exam psychosocial assessment and support. Abse of guidelines on mental health screening. Priority of physical health issues over comp psychosocial issues. Physical layout of units women-baby dyads per postnatal room) an small communities mean lack of privacy an confidentiality for sensitive discussions.
Shea et al (2011)[5]	Canada	To examine whether the implementation of a reminder system improved screening rates.	Intervention, quantitative, medical record review	Yes	Element of PNC: Postpartum screening for type 2 diabetes for women with GDM at 3 months after birth	Hospitals	**B:** Poor communication between obstetrici and primary care provider; providers uncer about screening recommendations; patien unaware of the risk of not screening, patie missing screening appointments due to competing time commitments.<br>**F:** Greater number of contacts with health providers increases screening rates.

First author (year of publication), reference	Country	Study objective(s)	Study design, methods, population/source of data	Inter-vention	Topic (routine, one element, specific target group)	PNC setting	Barriers (B) and facilitators (F) identifie
Bick et al (2012)[6]	UK	To assess if an intervention was associated with improved breastfeeding and maternal health outcomes, and enhanced women's views of care.	Intervention, quantitative, postpartum women	Yes	Element of PNC: Inpatient postnatal support for breastfeeding	Hospitals (large tertiary hospital)	**F:** 'Doing the right thing at the right time' approach: recognition of the need to targe content and timing of care and provision o information offered as a continuum across pregnancy, birth and the postnatal period. Support for breastfeeding commenced wit antenatal information, with subsequent cli care and processes to support breastfeedi promoted immediately following birth, wit midwives on the labour ward asked not to transfer women for at least two hours follo the birth to enable women to have longer 'quiet' time with their babies to initiate ski skin care.
Clark et al (2012)[7]	Multi-country		Literature review	No	Element of PNC: Screening for diabetes 6 weeks up to 6-12 months postpartum	Multiple	**B:** Discrepancy in guideline recommendati for postpartum screening. Fragmentation c medical care from the prenatal to postpart period contributes to lack of screening as t women are transitioned from obstetrical c and/or specialist care during the pregnanc and returned to their primary-care provide the postpartum period (obstetrical provide takes responsibility for the postpartum visi may not be perceived as being responsible diabetes screening postpartum, lack of communication toward primary care provi Lack of health insurance coverage (benefit at 6 weeks postpartum, impedes access to screening afterwards). Logistics of the OGT test (fasting and long time) means it is infrequently carried out in routine practice

First author (year of publication), reference	Country	Study objective(s)	Study design, methods, population/source of data	Inter-vention	Topic (routine, one element, specific target group)	PNC setting	Barriers (B) and facilitators (F) identifie
		To assess the rationale for screening postpartum and the barriers that have led to low screening rates.					Women do not return for postpartum scree and this particularly affects women at highe risk of T2DM. Barriers include lack of aware of the need for the test, a perception of go health and not needing further care, afraid being diagnosed with diabetes, negative experiences with care received during their pregnancy, and time and logistics factors to attend an OGTT.<br><br>**F:** Reminders and scheduled visits to health providers postpartum can improve postpar testing rates.
Hansen et al (2012)[8]	Norway	To describe the challenges, failures, strategies, and successes in implementing the baby-friendly hospital initiative in Norway.	Observational, mixed-methods, postpartum women	No	Element of PNC: Implementation of baby-friendly hospital initiative	Hospitals	**B:** Absorption of new knowledge to experienced staff and change established routines was a challenge; changes instigate externally seen as easier to accomplish; sor mothers did not want 24-hour rooming-in.<br><br>**F:** 24h rooming-in being voluntary (based o mother's choice)
Jolly et al (2012)[9]	UK	To assess the effectiveness of a peer support worker service on breast-feeding continuation.	Intervention, quantitative, postpartum women and medical record review	Yes	Element of PNC: Peer support for breastfeeding at 6 weeks and 6 months postpartum	Community/primary	**B:** Timing of peer support, nature and inter of peer support, levels of breastfeeding and support within women's social group

First author (year of publication), reference	Country	Study objective(s)	Study design, methods, population/source of data	Inter-vention	Topic (routine, one element, specific target group)	PNC setting	Barriers (B) and facilitators (F) identifie
Tawfik et al (2014)[10]	Afghanistan	To describe the value of applying modern quality improvement methods to improve service quality and to facilitate the integration of health services in a resource-constrained setting.	Intervention, mixed methods, medical records and postpartum women	Yes	Element of PNC: integration of family planning in postpartum care	Hospitals	**B:** Lack of private space for family planning counselling in the postpartum ward; Inabili some postpartum clients to decide on using contraception without consulting their husbands or mothers-in-law; Limited family planning counselling skills of postpartum nu and midwives (not able Involvement of hos leadership was essential to provide IUDs, fo example); Insufficient access to a variety of contraceptive methods at the postpartum ward. Public hospitals had all contraceptive commodities but smaller private hospitals not have all. **F:** Involvement of hospital leadership was essential.
Nithianandan et al (2016)[11]	Australia	To investigate barriers and enablers to implementing evidence-based, nationally recommended perinatal mental health screening and to inform sustainable implementation of a screening and referral programme, in women of refugee background.	Observational, Qualitative, healthcare providers	No	Element of PNC for a specific population: Postpartum mental health screening among refugee women	Multiple	**B:** Lack of availability of in-person interpret EPDS versions not available translated to ot languages; time constraints and capacity of mental health services. **F:** Staff training; inter-disciplinary roles to support referral; clearly communicated rob referral pathways which describe continuit care; female interpreters and healthcare professionals; social support during follow care.
Ortiz et al (2016)[12]	USA	To examine medical records of women with GDM to determine the proportion who	Observational, quantitative,	No	Element of PNC: Postpartum care	Hospitals (large tertiary hospital)	**B:** Lack of provider knowledge; lack of wom coming to postpartum visits (race/ethnicity

First author (year of publication), reference	Country	Study objective(s)	Study design, methods, population/source of data	Inter-vention	Topic (routine, one element, specific target group)	PNC setting	Barriers (B) and facilitators (F) identifie
		received a postpartum visit and recommended postpartum glucose screening, and the use of the 5 A's framework to provide preventive follow-up care.	medical record review		practices for women with GDM		ability to pay); lack of documentation in pa records of what was discussed
Weiss-Laxer et al (2016)[13]	USA and Greece	To describe recently published models and their core components, discuss barriers and facilitators to implementing such models, and highlight future practices and research needed to reduce the effects of maternal depression on mothers, families, and children.	Literature review	No	Element of PNC: Screening and management of maternal depression in pediatric services	Primary (pediatric)	**B:** Billing for maternal mental health treatn in pediatric primary care and funding for co located care coordinators, case managers, mental health providers are major systems level barriers to effective implementation.<br><br>**F:** Training of providers; availability of in ho mental health services; effective referral tracking systems; engaging physicians and/ mothers in meaningful education; expectin universal screening with easy access to screeners; and easy link to co-located or community-based care managers or social workers to facilitate a smooth referral process.
Lind et al (2017)[14]	USA	To evaluate the implementation of a postnatal PPD screening and treatment initiation process to identify and treat women at risk for postnatal PPD in a large	Intervention, quantitative, medical record review	Yes	Element of PNC: mental health screening and treatment from birth to 4 months postpartum	Primary (clinics in a multispecialty health care organisation)	**B:** No data linkage between mother's and newborn electronic medical records made difficult for clinicians to enter EPDS scores during well-child visits into the woman's medical record. Inability to record discussio about recommendations and treatment op with women with high EPDS scores if the

First author (year of publication), reference	Country	Study objective(s)	Study design, methods, population/source of data	Inter-vention	Topic (routine, one element, specific target group)	PNC setting	Barriers (B) and facilitators (F) identifie
		multispecialty health care organization.					woman declined treatment (no record generated).<br><br>**F:** Clinic staff were trained on how to link th mother and baby charts to facilitate easier documentation in the woman's chart durin well-child visits. Reminders of processes to records during regular staff meetings and v email, but in the long term integration of screening and treatment initiation informat into fields that are retrievable through electronic queries to facilitate review is nee
Niela-Vilen et al (2017)[15]	Finland	To explore parent–infant closeness and separation, and which factors promote closeness or result in separation in the birthing unit in the first 2hours after birth from the point of view of staff members.	Observational, Qualitative, healthcare providers (midwives and auxiliary nurses)	No	Element of PNC: parent-infant bonding in health facility in first 2 hours postpartum	Hospitals	**B:** Infants were separated from their paren some point for routine measurements to ensure the infants' health and well being (weight, length, head circumference, temperature, and oxygen saturation were measured routinely in each infant after the contact with the mother and breastfeeding usually performed in the same room and parent-infant closeness promoted by placin infant skin-to- skin again while these measurements were completed. babies wh needed an intervention were separated fro parents more often. The mother was perce as a threat to the infant's well-being if had fatigue, exhaustion). Both closeness and separation events were mainly controlled b staff members but in a few cases the participants described separation events

First author (year of publication), reference	Country	Study objective(s)	Study design, methods, population/source of data	Inter-vention	Topic (routine, one element, specific target group)	PNC setting	Barriers (B) and facilitators (F) identifie
							initiated by the parents.<br>**F:** Healthy newborn infants were allowed t<br>have immediate skin-to-skin for at least for<br>first hour, preferably until the first<br>breastfeeding was accomplished (it is a rou<br>care practice if the infant was healthy, awa<br>and in good condition; it was considered<br>necessary for the infants' well-being).
Fedock et al (2018)[16]	USA	To investigate predictors of universal screening and guideline-congruent care for perinatal depression by obstetrician-gynecologists and examined differences in practices with pregnant and postpartum patients.	Observational, Quantitative, healthcare providers (obstetric providers)	No	Element of PNC: Perinatal depression screening	Hospitals	**F:** Availability of on-site mental health servi<br>coordination with mental health service;<br>familiarity with a mental health profession;<br>provider's comfort with diagnosing depress<br>Providing guideline-congruent care was<br>associated with having time for follow-up<br>appointments with depressed patients and<br>clinical priority to address postpartum<br>depression.
Harvey et al (2018)[17]	Australia	To describe the development and evaluation of a community model for perinatal mental health based on the practice principles of: nurse-led; partnership approach; individualised evidenced based treatments and accessible, flexible service delivery.	Intervention, quantitative, postpartum women	Yes	Element of PNC: community model for perinatal mental health (up to 6 appointments)	Community	**F:** Appointments available in easy to access<br>welcoming, community locations; mental<br>health nurses have experience establishing<br>therapeutic relationship and are skilled in<br>providing holistic care

First author (year of publication), reference	Country	Study objective(s)	Study design, methods, population/source of data	Inter-vention	Topic (routine, one element, specific target group)	PNC setting	Barriers (B) and facilitators (F) identifie
January et al (2018)[18]	Zimbabwe	To determine the existing and potential opportunities and obstacles to screening for perinatal depression among women in Zimbabwe.	Literature review	No	Element of PNC: mental health screening	Community and primary care settings	**B:** Low use of PNC means fewer opportunit to provide mental health screening. Staff shortages and heavy workload at primary l stigma of reporting mental health illness, p referral of mental health illness. Lack of validated screening tools in languages othe than Shona.<br><br>**F:** Start perinatal mental health screening during antenatal care given high use in Zimbabwe). To address time constraints du screening for depression, could adopt shor versions of screening instruments such as t two-item Patient Health Questionnaire (PH which is administered as an initial brief screening for depression. Using community based screening in addition to health-facili based screening could overcome low use o facility-based PNC.
Talbot et al (2018)[19]	UK	To explore GPs' views and experiences of using the postnatal check as a health-related behaviour change opportunity.	Observational, quantitative, healthcare providers (general practitioners)	No	Element of PNC: long-term life style behaviour change 6-8 weeks postpartum	Primary (GPs)	**B:** Women are overwhelmed during the postpartum period for this type of informa GPs do not have enough time to cover life- behaviour change, GPs do not have the rig skills to provide it and do not think it is a g use of their time, GPs believed that other health professionals who have longer conti of care exposure to women were in a bette position to facilitate behaviour change.

First author (year of publication), reference	Country	Study objective(s)	Study design, methods, population/source of data	Inter-vention	Topic (routine, one element, specific target group)	PNC setting	Barriers (B) and facilitators (F) identifie
							**F:** Women going through a major change su as having a baby may be more motivated a supported to implement lifestyle changes, which may have been initiated during pregnancy, such as changes in smoking and drinking behaviours. GPs suggested differe healthcare professionals were in a better position to discuss behaviour change and specifically named health visitors, health trainers, midwives, dieticians, and specialis nurses as potential candidates.
McCauley et al (2019)[20]	Ghana	To investigate the knowledge, attitudes and perceptions of routine screening for maternal mental health during and after pregnancy among healthcare providers providing routine maternity care in Accra, Ghana.	Observational, Qualitative, healthcare providers (doctors and nurse-midwives)	No	Element of PNC: mental health screening	Hospitals (tertiary)	**B:** Lack of time for mental health assessme healthcare staff shortages, staff not trained assess and manage maternal mental health cultural stigmas surrounding mental health **F:** Healthcare providers' awareness of men health in general and the understanding th women were at high risk of mental health disorders during and after pregnancy.
Taylor et al (2019)[21]	Australia	To explore the role of clinical facilitation in implementing and sustaining a perinatal and infant telemental health service.	Observational, Qualitative, healthcare providers	No	Element of PNC: mental health screening and treatment (telemedicine)	Multiple	**B:** Rural, sparse population cannot sustain specialist mental health services and this re in service gaps. Transient workforce and funding issues. Perinatal and infant mental health is a highly specialised, low-volume service. Relative to other telehealth service this reduces the ability to embed the servic routine service delivery. The intermittent nature of such a service means that local he providers may not refer appropriate patien

First author (year of publication), reference	Country	Study objective(s)	Study design, methods, population/source of data	Inter-vention	Topic (routine, one element, specific target group)	PNC setting	Barriers (B) and facilitators (F) identifie
							the service as they do not know or forget th service is available.<br><br>**F:** Mental health workers who had used telehealth intervention saw benefits such a allowing expert input (rather than generalis adult clinicians) into care planning, providi the capacity for patients to be managed clo to home, upskilling the remote workforce, reducing professional isolation and providi sense of security for remote care providers Regular contact(in-person and online) with clinical facilitator was described as helping maintain service providers' awareness of th existence of this service.
Thomson et al (2019)[22]	UK	To explore afterbirth provision for women who have had a traumatic/distressing birth in NHS hospital trusts in England.	Observational, quantitative, healthcare trusts (NHS)	No	Element of PNC: debriefing after traumatic birth	Hospitals: Hospital trusts - tertiary (NHS)	**B:** No specific funding for the service, which not offered to women proactively, workloa constraints<br><br>**F:** Independence of NHS trusts - pursuing service provision based on women's needs, feedback on maternity services from wome
**Topic: Postnatal care for specific population**							
Saias et al (2012)[23]	France	To evaluate fidelity in a 27-month program addressing maternal and child health	Observational, Quantitative, healthcare	No	PNC for a specific population: Postnatal home visits for vulnerable families	Community	**B:** The negative impact of the family's socia environment - home visitors had difficultie organizing home visits and intervening according to the guideline. The frequency o

First author (year of publication), reference	Country	Study objective(s)	Study design, methods, population/source of data	Inter-vention	Topic (routine, one element, specific target group)	PNC setting	Barriers (B) and facilitators (F) identifie
		which took place in France between 2006 and 2011.	providers' case notes				visits and the structure of each visit were disrupted by the social situation of the fam (difficulty maintaining attention and discus visit objectives), due in part to unpredictab living conditions. Difficulties in maintaining relationship with families.
Hannan (2013)[24]	USA	To examine the effects of a low cost advanced practice nurse telephone intervention for 2 months post birth in low-income first time mothers with healthy full term infants.	Intervention, quantitative, postpartum women	Yes	PNC for a specific population: Routine postpartum support for low-income mothers up to 2 months post-discharge	Hospital -> Community discharge	**F:** Language options to improve communic with mothers
Cegolon et al (2020)[25]	Italy	To investigate the impact of the outstanding factors on length of stay following caesarean section, with the view of providing epidemiological figures potentially useful to support the design and evaluation of obstetric care policies in an Italian region.	Observational, quantitative, medical record review	No	PNC for a specific population: care for women post caesarean section before discharge from hospital	Hospitals	**B:** Hospital costs
**Topic: Routine package of postnatal care**							

First author (year of publication), reference	Country	Study objective(s)	Study design, methods, population/source of data	Inter-vention	Topic (routine, one element, specific target group)	PNC setting	Barriers (B) and facilitators (F) identifie
Forster et al (2006)[26]	Australia	To describe staffing and related issues in the provision of postnatal care in public hospitals in Victoria from the caregivers' perspective; and to explore what impact (if any) providers believe staffing issues have on their ability to provide high quality care to women.	Observational, mixed-methods, healthcare providers (midwives)	No	Routine PNC: Hospital-based pre-discharge care	Hospitals (multiple types)	**B:** Staffing mix/numbers; patient mix; the impact of staff leave (both planned and unplanned); and staff/patient ratios. Fragmented postnatal care if provided by n midwives (other cadres are not qualified to carry out the full range of activities). Use of agency staff: nursing agencies do not have insurance to cover their staff working in bir suite or antenatal areas, meaning agency st is more likely to be posted in postnatal war rather than other maternity areas. Student midwives may help with staffing levels in so instances, but at other times may increase workload for midwives on postnatal wards. Birth suite was prioritised over the postnat ward during busy periods. Staff/patient rat the area of postnatal care were inadequate especially due to rise in needs for example, csection births.<br><br>**F:** Problems with staff recruitment and retention could be addressed with higher financing of registered nurses to undertake Postgraduate Diploma in Midwifery and providing clinical placements for students

First author (year of publication), reference	Country	Study objective(s)	Study design, methods, population/source of data	Inter-vention	Topic (routine, one element, specific target group)	PNC setting	Barriers (B) and facilitators (F) identifie
Schmied et al (2009)[27]	Australia	To design, implement and evaluate strategies to improve the quality and content of hospital-based postnatal care.	Intervention, quantitative, postpartum women	Yes	Routine PNC: Hospital-based pre-discharge care	Hospitals	**F:** More intensive training in communicatio and working in partnership with women an families may increase midwives' skills
Hannan (2016)[28]	USA	To test a nurse practitioner intervention using cell phone and texting on maternal/infant outcomes.	Intervention, quantitative, postpartum women	Yes	Routine PNC: continuity of care in first 6 months postpartum using nurse practitioners	Community (after discharge from hospital)	**B:** Financial constraints lead to reducing ma community health care services, especially those in maternal child health. Different lev insurance coverage between children espe low levels among migrants and poor familie contributes to lower postnatal care.
Rumbold et al (2010)[29]	Australia	To describe patterns of the delivery of maternity care and service gaps on a broad scale, using data from baseline clinical audits in 34 Indigenous primary health centres participating in a national quality improvement intervention.	Observational, quantitative, medical record review	No	Routine PNC: assessment of adherence to protocols/ guidelines between 2-14 months after birth	Primary	**B:** Lack of adherence to recommended screening investigations; poor documentat of advice regarding health risk factors durin the postnatal period
Bick et al (2011)[30]	UK	To present data on the views of midwives from one large maternity unit in the South of England following the introduction of an organisation wide quality improvement initiative to improve in-patient	Observational, mixed-methods, healthcare providers (midwives)	Yes	Routine PNC: in-patient postnatal care and processes to transfer women home	Hospitals (large tertiary hospital)	**B:** Additional workload the new postnatal record generated for midwives. The previo notes used at the unit did not require midv to document a care plan for the woman an infant or provide guidance on details of ca be covered at specific time periods. New fo were time consuming to complete, especia capture the additional information require plan care during the first home visit (extra

First author (year of publication), reference	Country	Study objective(s)	Study design, methods, population/source of data	Inter-vention	Topic (routine, one element, specific target group)	PNC setting	Barriers (B) and facilitators (F) identifie
		postnatal care and processes to transfer women home.					needed if the previous midwife who had ca for a woman had not completed all relevan sections of the notes, for example hospital midwife not including obstetric history).<br><br>**F:** New forms were perceived as providing better support for less experienced staff. A engagement of health professionals is one the conditions to support implementation such organisational change. A solution to duplication of notes could be introducing a of notes for the whole maternity episode.
Beake et al (2012)[31]	UK	To assess the impact of NICE guidelines for routine postnatal care and identify how to improve the continuum of care.	Observational, mixed-methods, healthcare providers, women and review of clinic activities	Yes	Routine PNC: Hospital-based pre-discharge care	Hospitals (maternity unit)	**B:** Physical care women needed was not provided (e.g. infections were overlooked), linked to lack of communication and wome not being listened to.<br><br>**F:** Women and babies staying longer (up to hours) in the labour ward to facilitate skin t skin before being moved to the postnatal w based on women's request, maternity supp workers organised sessions in the ward covering bathing and skincare. Visual aids developed for parents for use in hospital ar home

First author (year of publication), reference	Country	Study objective(s)	Study design, methods, population/source of data	Inter-vention	Topic (routine, one element, specific target group)	PNC setting	Barriers (B) and facilitators (F) identifie
Rayner et al (2013)[32]	Australia	To explore care providers' views and experiences of postnatal care in private hospitals.	Observational, mixed-methods, healthcare providers	No	Routine PNC: Provision in private hospitals	Hospitals (private)	**B:** Standard care plans have a lot of items a they are not individualised to women's nee staffing challenges - lack of midwives mean non-midwife cadres providing PNC; lack of continuity of care (staff shortages and man midwives working part-time); and the impa key players in postnatal care (including visi while important for women, they make wo tired and there is chaos on the ward, mana especially if they feel that managers do not prioritise PNC, and obstetricians- perceive role as minimal in PNC, mostly "social"). Women might have an expectation that ba will be in a nursery and midwives are confli because they support rooming-in.
Haran et al (2014)[33]	Multi-country (3 HICs)	To compare the scope and content, and assess the quality of clinical guidelines about routine postpartum care in primary care.	Guideline review, mixed-methods evidence synthesis	No	Routine PNC: review of clinical guidelines in primary care	Primary	**B:** Lack of evidence on timings of routine vi **F:** More mother-centred approach to the ti of visits rather than having a standardised v schedule
McMahon et al (2015)[34]	Tanzania	To present findings on time to discharge, characteristics of women who depart early, receipt of postpartum messaging and the experience of post-delivery facility departure as described by women in rural Tanzania.	Observational, mixed-methods, postpartum women and community members	No	Routine PNC: Hospital-based pre-discharge care	Multiple facility types	**B:** Short postnatal length of stay in facility v related to: women's personal expectations provider encouragement to leave and an understanding that an early discharge frees space for others and is more comfortable f mother-baby pairs; inability to obtain wate prepare foods as well as excessive noise in facility. Women needed to care for other

First author (year of publication), reference	Country	Study objective(s)	Study design, methods, population/source of data	Inter-vention	Topic (routine, one element, specific target group)	PNC setting	Barriers (B) and facilitators (F) identifie
							children at home and leave when transportation was available.<br><br>**F:** Provider knowledge on optimal stays aft facility delivery, conditions and services in facilities should be improved, increasing th facility staffing and encouraging compassio the part of health professionals.
Forster et al (2016)[35]	Australia	To explore the structure and organisation of public hospital home-based postnatal care in Victoria, Australia.	Observational, mixed-methods, hospitals (maternity unit managers)	No	Routine PNC: postnatal home-based care after hospital discharge	Hospitals (tertiary public)	**B:** Staffing of home-based PNC: staffing adequacy related to difficulties meeting the fluctuating demand at short notice for the service and the need (and ability) to be flex Transport: a lack of hospital cars and issue regarding reimbursement for staff that use their own cars. Safety of healthcare provide conducting home visits. Women declining h visits. Women living outside the home-base service boundaries, with no other services accessible. Staff rarely work on PNC alone; one-third of hospitals employ midwives to solely in home-based postnatal care. Staffin across the continuum of care is likely to be aimed at increasing continuity and/or encouraging midwives to work across their scope of practice, but may contribute to a lower priority given to postnatal care and t stress of the midwife, factors which have b identified as obstacles to 'good' postnatal c

First author (year of publication), reference	Country	Study objective(s)	Study design, methods, population/source of data	Inter-vention	Topic (routine, one element, specific target group)	PNC setting	Barriers (B) and facilitators (F) identifie
							**F:** Dedicated staff for the home-based post care service (or for postnatal care in hospit and at home) meant higher continuity of ca and less inconsistency in advice. Designatec coordinator for the home-based postnatal service.
Haskins et al (2016)[36]	South Africa	To investigate the provision of MCWH services at PHC well-child clinics in one district in KwaZulu-Natal (KZN) and to describe a detailed picture of service delivery at PHC level.	Observational, mixed-methods, healthcare providers, women and review of clinic activities	No	Routine PNC: continuity of care at primary health care level up to 1 year postpartum	Primary	**B:** Equipment to provide all the services wa available but frequently scattered around t clinic and not available in the rooms where health workers were providing a particular service. Medications, including oral contraceptives and antiretroviral drugs, we kept with the professional nurse in all clinic Guidance to users: upon arrival at the clinic mothers and babies seeking well-child serv received directions from a staff member. Although these directions determined the pathway by which mother–baby pairs proceeded through the clinic and, therefore which services they could access, directions were given in an ad hoc way, usually by a ju member of the clinic staff (nurse, nursing assistants, CHWs, receptionist, security gua and without clear guidance. Mothers who w HIV positive were directed to the HIV couns before going to immunisation room where enrolled nurse administered the immunisat HIV-positive mothers often left the clinic without being seen by a professional nurse thus bypassing essential postnatal PMTCT

First author (year of publication), reference	Country	Study objective(s)	Study design, methods, population/source of data	Inter-vention	Topic (routine, one element, specific target group)	PNC setting	Barriers (B) and facilitators (F) identifie
							services. HIC negative mothers were direct the newborn immunisation room where th immunisation was administered after whic mother left the clinic without being seen b professional nurse. Mothers had limited opportunity to privately consult with health workers during the clinic visit; lack of priva during visit (newborn weighing) is a proble and also a barrier to private conversations taking place about mother's health.
Miller et al (2016)[37]	Multi-country	To present a systematic review of evidence-based clinical practice guidelines for routine antenatal, intrapartum, and postnatal care, categorising them as recommended, recommended only for clinical indications, and not recommended.	Guideline review, mixed-methods evidence synthesis	No	Routine PNC: Review of guidelines and quality of maternity care (including PNC)	Multiple, mainly facility-based	**B:** Adherence to guidelines is a problem du inconsistency of guidelines, which can be d contextual adaptation, but can be detrime this casts doubt on evidence.<br><br>**F:** Note that studies of guideline implementation targeting provider adhere alone are unlikely to succeed without a sys approach and engagement of women and communities. Encourage research on how guidelines are understood and applied by e users, and why conflicting guidelines arise.
Chukwuma et al (2017)[38]	Nigeria	To explore postnatal care referral behavior by TBAs in Nigeria, including the perceived factors that may deter or promote referrals to skilled health workers.	Observational, Qualitative, healthcare providers, Traditional birth	No	Routine PNC: postnatal care referral practices of traditional birth attendants	Community	**B:** TBAs did not offer postnatal care to clie following delivery, that is retaining her clie over at least one day and conducting regul assessments to detect complications. No T reported referring her clients to skilled hea workers for maternal postnatal care; refer formal care due to complications would ca

First author (year of publication), reference	Country	Study objective(s)	Study design, methods, population/source of data	Inter-vention	Topic (routine, one element, specific target group)	PNC setting	Barriers (B) and facilitators (F) identifie
			attendants (TBAs) and women				TBAs reputation to suffer. Two health work stated that TBAs in their communities left immediately after delivering the placenta a baby if the mother and neonate did not dev complications during delivery.<br><br>**F:** Incorporating TBAs into local health deci making committees and generally having a more cordial relationship between formal healthcare providers and TBAs could impro postnatal referral by TBAs to the facility aft delivery (for routine care and for complications). This could be incentivised, using non-financial incentives/prizes garne recognition for referrals; and supplies like diapers and mosquito nets to distribute to clients.
Perry et al (2017)[39]	Multi-country	To summarize the various approaches used by the programs, projects and studies whose effectiveness has been assessed and included in a comprehensive database.	Literature review	No	Routine PNC: review of effectiveness of community-based primary health care	Primary (community-based)	**B:** Context-specific resource availability, logistical challenges, contextual constraints (including health system).<br><br>**F:** Community engagement was essential element (the context must be carefully considered to select the most appropriate combinations of interventions and implementation strategies). Promptly selec and training new CHWs to replace those wl are no longer functioning in this capacity.

First author (year of publication), reference	Country	Study objective(s)	Study design, methods, population/source of data	Inter-vention	Topic (routine, one element, specific target group)	PNC setting	Barriers (B) and facilitators (F) identifie
Yee et al (2017)[40]	USA	To estimate whether postpartum visit attendance was improved in women exposed to a postpartum patient navigation program compared with those who received care immediately before the program's initiation and to assess whether other postpartum health behaviors improved during the intervention period.	Intervention, quantitative, postpartum women	Yes	Routine PNC: Continuity of care up to 12 weeks postpartum	Hospitals (tertiary)	**B:** Clinicians are too busy to help women navigate PNC.<br>**F:** Multidisciplinary feature to support navigation of PNC was staffed by non-physicians; use of text messaging to align w patient preferences.
Pallangyo et al (2018)[41]	Tanzania	To explore strategies used in a facilitation intervention to improve postpartum care (IPPC) in a low-resource suburb in Dar es Salaam, Tanzania.	Intervention, qualitative, health facility teams	Yes	Routine PNC: in-facility care (inpatient and outpatient)	Multiple (primary, secondary and tertiary health facilities)	**B:** Inadequate resources, unclear organizat of physical structure, and lack of clarity abo how to improve postpartum care among interdisciplinary teams.<br><br>**F:** Raising awareness and knowledge of postpartum care among healthcare provide through training, meetings, and clinical pra builds confidence and created empowerme and agents of change and this was central t the mobilization of resources related to staffing, space, and equipment. Educationa displays produced from locally available materials to display key contents of the guidelines (in spaces where providers and women can see them). Improvement of the documentation of care and communicatior

First author (year of publication), reference	Country	Study objective(s)	Study design, methods, population/source of data	Inter-vention	Topic (routine, one element, specific target group)	PNC setting	Barriers (B) and facilitators (F) identifie
							among teams within and between instituti (including referral case notes).
Grylka-Baeschlin et al (2020)[42]	Switzerland	To compare characteristics of users of a midwifery network which referred women to outpatient postpartum care providers with those of women organising care themselves. Additionally, we investigated benefits of the network for women and health professionals.	Observational, mixed-methods, postpartum women and healthcare providers (midwives)	Yes	Routine PNC: Effect of PNC organised through a midwifery network (particularly for socially disadvantaged families)	Primary	**B:** Women's perspectives: Some women we very frustrated trying to organise out of hospital postpartum care themselves; inabi to choose preferred midwife within midwif network (other women did not have concre ideas or expectations about the midwife ar were not challenged with the impersonal organisation of care); accessibility and relia of the midwife (women became nervous if was difficult to reach the midwife or if she not adhere to meeting times and was late). Midwives' perspectives: introduction of ne technologies (mobile application); imbalan workload due to unpredictability of births; language difficulties and would have appreciated the support of interpreters wit cultural mediation skills.<br><br>**F:** Women's perspectives: Clarity on organisation of the out of hospital postpart care (PNC through midwifery network was and straightforward and saved time, worrie and stress); email communication with mid was appreciated as very friendly and

First author (year of publication), reference	Country	Study objective(s)	Study design, methods, population/source of data	Inter-vention	Topic (routine, one element, specific target group)	PNC setting	Barriers (B) and facilitators (F) identifie
							professional; women with migration background recognised the midwife as a cultural mediator. Midwives' perspectives: mobile application facilitated their work organisation; possibility to choose clients c to the midwife's home or to other patients therefore to keep route short (logistics); ho visits allow deep insight into family lives an individualised support with vulnerabilities observed. The midwifery network interface with the hospitals about the follow-up supp offered to families and could be extended t relay feedback to the hospital about wome and their children who, according to midwi assessment, left the hospital in a poor state health.
Krishnamurti et al (2020)[43]	USA	To analyze the tradeoffs that providers may make to lend insight into which elements of care those providers may interpret as less valuable or find more challenging to administer given their time constraints. To explore providers' support for and capability of performing this care using telemedicine.	Observational, Quantitative, healthcare providers (gynecologists, family medicine physicians, nurses and midwives)	No	Routine PNC: assessment of adherence to protocols/guidelines	Multiple	**B:** Time constraints during clinical visits

References

1. Steele AM, Beadle M. A survey of postnatal debriefing. *J Adv Nurs* 2003;43(2):130-6. doi: 10.1046/j.1365-2648.2003.02687.x [published Online First: 2003/07/02]
2. Buist A, Condon J, Brooks J, et al. Acceptability of routine screening for perinatal depression. *J Affect Disord* 2006;93(1-3):233-7. doi: 10.1016/j.jad.2006.02.019 [published Online First: 2006/05/02]
3. Tappin D, Britten J, Broadfoot M, et al. The effect of health visitors on breastfeeding in Glasgow. *Int Breastfeed J* 2006;1:11. doi: 10.1186/1746-4358-1-11 [published Online First: 2006/07/11]
4. Yelland J, McLachlan H, Forster D, et al. How is maternal psychosocial health assessed and promoted in the early postnatal period? Findings from a review of hospital postnatal care in Victoria, Australia. *Midwifery* 2007;23(3):287-97. doi: 10.1016/j.midw.2006.06.003 [published Online First: 2006/11/23]
5. Shea AK, Shah BR, Clark HD, et al. The effectiveness of implementing a reminder system into routine clinical practice: does it increase postpartum screening in women with gestational diabetes? *Chronic Dis Can* 2011;31(2):58-64. [published Online First: 2011/04/07]
6. Bick D, Murrells T, Weavers A, et al. Revising acute care systems and processes to improve breastfeeding and maternal postnatal health: a pre and post intervention study in one English maternity unit. *BMC Pregnancy Childbirth* 2012;12:41. doi: 10.1186/1471-2393-12-41 [published Online First: 2012/06/08]
7. Clark HD, Keely E. Getting mothers with gestational diabetes to return for postpartum testing: What works and what does not. *Diabetes Management* 2012;2(1):33.
8. Hansen MN, Bærug A, Nylander G, et al. Challenges and successes: the Baby-Friendly Initiative in Norway. *J Hum Lact* 2012;28(3):285-8. doi: 10.1177/0890334412444162 [published Online First: 2012/06/23]
9. Jolly K, Ingram L, Freemantle N, et al. Effect of a peer support service on breast-feeding continuation in the UK: a randomised controlled trial. *Midwifery* 2012;28(6):740-5. doi: 10.1016/j.midw.2011.08.005 [published Online First: 2011/09/29]
10. Tawfik Y, Rahimzai M, Ahmadzai M, et al. Integrating family planning into postpartum care through modern quality improvement: experience from Afghanistan. *Glob Health Sci Pract* 2014;2(2):226-33. doi: 10.9745/ghsp-d-13-00166 [published Online First: 2014/10/03]
11. Nithianandan N, Gibson-Helm M, McBride J, et al. Factors affecting implementation of perinatal mental health screening in women of refugee background. *Implement Sci* 2016;11(1):150. doi: 10.1186/s13012-016-0515-2 [published Online First: 2016/11/20]
12. Ortiz FM, Jimenez EY, Boursaw B, et al. Postpartum Care for Women with Gestational Diabetes. *MCN Am J Matern Child Nurs* 2016;41(2):116-22. doi: 10.1097/nmc.0000000000000215 [published Online First: 2016/02/26]
13. Weiss-Laxer NS, Platt R, Osborne LM, et al. Beyond screening: a review of pediatric primary care models to address maternal depression. *Pediatr Res* 2016;79(1-2):197-204. doi: 10.1038/pr.2015.214 [published Online First: 2015/10/21]
14. Lind A, Richter S, Craft C, et al. Implementation of Routine Postpartum Depression Screening and Care Initiation Across a Multispecialty Health Care Organization: An 18-Month Retrospective Analysis. *Matern Child Health J* 2017;21(6):1234-39. doi: 10.1007/s10995-017-2264-5 [published Online First: 2017/02/06]
15. Niela-Vilén H, Feeley N, Axelin A. Hospital routines promote parent-infant closeness and cause separation in the birthing unit in the first 2 hours after birth: A pilot study. *Birth* 2017;44(2):167-72. doi: 10.1111/birt.12279 [published Online First: 2017/02/16]

16. Fedock GL, Alvarez C. Differences in Screening and Treatment for Antepartum Versus Postpartum Patients: Are Providers Implementing the Guidelines of Care for Perinatal Depression? *J Womens Health (Larchmt)* 2018;27(9):1104-13. doi: 10.1089/jwh.2017.6765 [published Online First: 2018/05/15]
17. Harvey ST, Bennett JA, Burmeister E, et al. Evaluating a nurse-led community model of service for perinatal mental health. *Collegian* 2018;25(5):525-31.
18. January J, Chimbari MJ. Opportunities and obstacles to screening for perinatal depression among women in Zimbabwe: A narrative review of literature. *S Afr J Psychiatr* 2018;24:1127. doi: 10.4102/sajpsychiatry.v24i0.1127 [published Online First: 2018/09/29]
19. Talbot H, Strong E, Peters S, et al. Behaviour change opportunities at mother and baby checks in primary care: a qualitative investigation of the experiences of GPs. *Br J Gen Pract* 2018;68(669):e252-e59. doi: 10.3399/bjgp18X695477 [published Online First: 2018/03/14]
20. McCauley M, Brown A, Ofosu B, et al. "I just wish it becomes part of routine care": healthcare providers' knowledge, attitudes and perceptions of screening for maternal mental health during and after pregnancy: a qualitative study. *BMC Psychiatry* 2019;19(1):279. doi: 10.1186/s12888-019-2261-x [published Online First: 2019/09/11]
21. Taylor M, Kikkawa N, Hoehn E, et al. The importance of external clinical facilitation for a perinatal and infant telemental health service. *J Telemed Telecare* 2019;25(9):566-71. doi: 10.1177/1357633x19870916 [published Online First: 2019/10/22]
22. Thomson G, Garrett C. Afterbirth support provision for women following a traumatic/distressing birth: Survey of NHS hospital trusts in England. *Midwifery* 2019;71:63-70. doi: 10.1016/j.midw.2019.01.004 [published Online First: 2019/01/29]
23. Saïas T, Lerner E, Greacen T, et al. Evaluating fidelity in home-visiting programs a qualitative analysis of 1058 home visit case notes from 105 families. *PLoS One* 2012;7(5):e36915. doi: 10.1371/journal.pone.0036915 [published Online First: 2012/05/26]
24. Hannan J. APN telephone follow up to low-income first time mothers. *J Clin Nurs* 2013;22(1-2):262-70. doi: 10.1111/j.1365-2702.2011.04065.x [published Online First: 2012/08/01]
25. Cegolon L, Mastrangelo G, Maso G, et al. Determinants of length of stay after cesarean sections in the Friuli Venezia Giulia Region (North-Eastern Italy), 2005-2015. *Sci Rep* 2020;10(1):19238. doi: 10.1038/s41598-020-74161-2 [published Online First: 2020/11/08]
26. Forster DA, McLachlan HL, Yelland J, et al. Staffing in postnatal units: is it adequate for the provision of quality care? Staff perspectives from a state-wide review of postnatal care in Victoria, Australia. *BMC Health Serv Res* 2006;6:83. doi: 10.1186/1472-6963-6-83 [published Online First: 2006/07/05]
27. Schmied V, Cooke M, Gutwein R, et al. An evaluation of strategies to improve the quality and content of hospital-based postnatal care in a metropolitan Australian hospital. *J Clin Nurs* 2009;18(13):1850-61. doi: 10.1111/j.1365-2702.2008.02746.x [published Online First: 2009/07/30]
28. Hannan J, Brooten D, Page T, Galindo A, Torres M. Low-Income First-Time Mothers: Effects of APN Follow-up Using Mobile Technology on Maternal and Infant Outcomes. Glob Pediatr Health. 2016 Jul 26;3:2333794X16660234. doi: 10.1177/2333794X16660234.
29. Rumbold AR, Bailie RS, Si D, et al. Assessing the quality of maternal health care in Indigenous primary care services. *Med J Aust* 2010;192(10):597-8. doi: 10.5694/j.1326-5377.2010.tb03646.x [published Online First: 2010/05/19]
30. Bick DE, Rose V, Weavers A, et al. Improving inpatient postnatal services: midwives views and perspectives of engagement in a quality improvement initiative. *BMC Health Serv Res* 2011;11:293. doi: 10.1186/1472-6963-11-293 [published Online First: 2011/11/03]
31. Beake S, Bick D, Weavers A. Revising care to meet maternal needs post birth: an overview of the hospital to home postnatal study. *Pract Midwife* 2012;15(6):10, 12-3. [published Online First: 2012/08/07]

32. Rayner JA, McLachlan HL, Peters L, et al. Care providers' views and experiences of postnatal care in private hospitals in Victoria, Australia. *Midwifery* 2013;29(6):622-7. doi: 10.1016/j.midw.2012.05.006 [published Online First: 2012/11/06]
33. Haran C, van Driel M, Mitchell BL, et al. Clinical guidelines for postpartum women and infants in primary care-a systematic review. *BMC Pregnancy Childbirth* 2014;14:51. doi: 10.1186/1471-2393-14-51 [published Online First: 2014/01/31]
34. McMahon SA, Mohan D, LeFevre AE, et al. "You should go so that others can come"; the role of facilities in determining an early departure after childbirth in Morogoro Region, Tanzania. *BMC Pregnancy Childbirth* 2015;15:328. doi: 10.1186/s12884-015-0763-1 [published Online First: 2015/12/15]
35. Forster DA, McKay H, Powell R, et al. The structure and organisation of home-based postnatal care in public hospitals in Victoria, Australia: A cross-sectional survey. *Women Birth* 2016;29(2):172-9. doi: 10.1016/j.wombi.2015.10.002 [published Online First: 2015/11/14]
36. Haskins LJ, Phakathi SP, Grant M, et al. Fragmentation of maternal, child and HIV services: A missed opportunity to provide comprehensive care. *Afr J Prim Health Care Fam Med* 2016;8(1):e1-e8. doi: 10.4102/phcfm.v8i1.1240 [published Online First: 2017/02/06]
37. Miller S, Abalos E, Chamillard M, et al. Beyond too little, too late and too much, too soon: a pathway towards evidence-based, respectful maternity care worldwide. *Lancet* 2016;388(10056):2176-92. doi: 10.1016/s0140-6736(16)31472-6 [published Online First: 2016/09/20]
38. Chukwuma A, Mbachu C, Cohen J, et al. "Once the delivery is done, they have finished": a qualitative study of perspectives on postnatal care referrals by traditional birth attendants in Ebonyi state, Nigeria. *BMC Pregnancy Childbirth* 2017;17(1):429. doi: 10.1186/s12884-017-1616-x [published Online First: 2017/12/21]
39. Perry HB, Sacks E, Schleiff M, et al. Comprehensive review of the evidence regarding the effectiveness of community-based primary health care in improving maternal, neonatal and child health: 6. strategies used by effective projects. *J Glob Health* 2017;7(1):010906. doi: 10.7189/jogh.07.010906 [published Online First: 2017/07/08]
40. Yee LM, Martinez NG, Nguyen AT, et al. Using a Patient Navigator to Improve Postpartum Care in an Urban Women's Health Clinic. *Obstet Gynecol* 2017;129(5):925-33. doi: 10.1097/aog.0000000000001977 [published Online First: 2017/04/07]
41. Pallangyo E, Mbekenga C, Olsson P, et al. Implementation of a facilitation intervention to improve postpartum care in a low-resource suburb of Dar es Salaam, Tanzania. *Implement Sci* 2018;13(1):102. doi: 10.1186/s13012-018-0794-x [published Online First: 2018/07/30]
42. Grylka-Baeschlin S, Iglesias C, Erdin R, et al. Evaluation of a midwifery network to guarantee outpatient postpartum care: a mixed methods study. *BMC Health Serv Res* 2020;20(1):565. doi: 10.1186/s12913-020-05359-3 [published Online First: 2020/06/24]
43. Krishnamurti T, Simhan HN, Borrero S. Competing demands in postpartum care: a national survey of U.S. providers' priorities and practice. *BMC Health Serv Res* 2020;20(1):284. doi: 10.1186/s12913-020-05144-2 [published Online First: 2020/04/08]
